# Optical and X-ray Fluorescent Nanoparticles for Dual Mode Bioimaging

**DOI:** 10.1021/acsnano.0c10127

**Published:** 2021-02-15

**Authors:** Giovanni M. Saladino, Carmen Vogt, Yuyang Li, Kian Shaker, Bertha Brodin, Martin Svenda, Hans M. Hertz, Muhammet S. Toprak

**Affiliations:** Department of Applied Physics, Biomedical and X-Ray Physics, KTH Royal Institute of Technology, SE 10691 Stockholm, Sweden

**Keywords:** core−shell nanoparticles, silica coated nanoparticles, fluorescent dye doping, contrast agent, bioimaging, X-ray fluorescence, XFCT

## Abstract

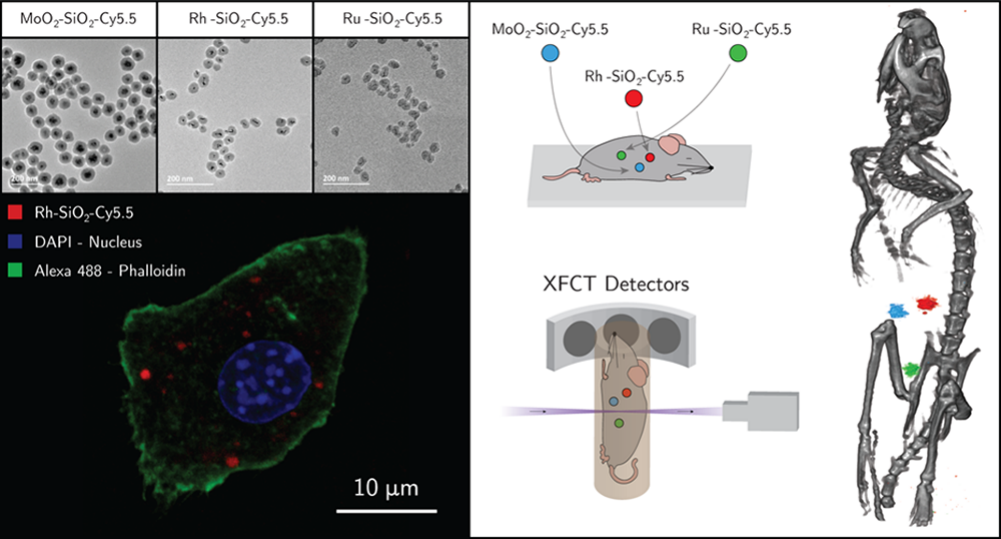

Nanoparticle (NP) based contrast agents detectable via different imaging modalities (multimodal properties) provide a promising strategy for noninvasive diagnostics. Core–shell NPs combining optical and X-ray fluorescence properties as bioimaging contrast agents are presented. NPs developed earlier for X-ray fluorescence computed tomography (XFCT), based on ceramic molybdenum oxide (MoO_2_) and metallic rhodium (Rh) and ruthenium (Ru), are coated with a silica (SiO_2_) shell, using ethanolamine as the catalyst. The SiO_2_ coating method introduced here is demonstrated to be applicable to both metallic and ceramic NPs. Furthermore, a fluorophore (Cy5.5 dye) was conjugated to the SiO_2_ layer, without altering the morphological and size characteristics of the hybrid NPs, rendering them with optical fluorescence properties. The improved biocompatibility of the SiO_2_ coated NPs without and with Cy5.5 is demonstrated *in vitro* by Real-Time Cell Analysis (RTCA) on a macrophage cell line (RAW 264.7). The multimodal characteristics of the core–shell NPs are confirmed with confocal microscopy, allowing the intracellular localization of these NPs *in vitro* to be tracked and studied. *In situ* XFCT successfully showed the possibility of *in vivo* multiplexed bioimaging for multitargeting studies with minimum radiation dose. Combined optical and X-ray fluorescence properties empower these NPs as effective macroscopic and microscopic imaging tools.

Nanoparticles (NPs) as contrast agents for different imaging modalities not only are the subject of intense research but also are already available as commercial products either for preclinical research or clinical imaging.^1,2^ Every imaging technique requires the use of specific contrast agents; for example, superparamagnetic iron oxides as magnetic nanomaterials for magnetic resonance imaging (MRI); quantum dots, gold and rare earth oxide NPs for optical imaging; silica (SiO_2_) NPs for ultrasound imaging; and radionuclide-labeled compounds for nuclear imaging (PET, SPECT). Furthermore, X-ray fluorescent NPs have been successfully employed in bioimaging, to highlight biophysical characteristics and features in cellular environments.^3,4^

Recently, we demonstrated the use of NPs as contrast agents for X-ray fluorescence computed tomography (XFCT) in preclinical research and for tumor detection using MoO_2_ NPs.^5,6^ Furthermore, the potential use of Rh and Ru based NPs as XFCT contrast agents has also been demonstrated.^7,8^ With an established library of potential contrast agents for XFCT, an increase in the functionality of the NPs is a natural step.

SiO_2_ is a biocompatible material, known for offering a versatile platform via its facile surface modification. Coating NPs with a SiO_2_ layer is already a proven method of modulating NPs’ toxicity in contact with biological systems. Furthermore, the SiO_2_ layer can also play the role of host to additional molecules with the possibility of increasing the functionality of the NP core–SiO_2_ shell entities.^9,10^ The introduction of multiple properties to the same structure constitutes a relevant and indispensable tool for biomedical applications.^11^

Cy5.5 is a near-infrared fluorophore ideal for *in vitro* and *in vivo* bioimaging applications, where background autofluorescence is a concern.^12^ The emission wavelength in the near-infrared allows a long penetration depth in tissue necessary for *in vivo* imaging, thus easing the excitation and detection in biological systems where autofluorescence is a concern. While the photostability of Cy5.5 alone is known to be poor, the SiO_2_ encapsulated dye molecules have been demonstrated to have increased brightness, extended photostability, and higher penetration depth up to 2 cm compared to the free dye.^13−15^ The Cy5.5-*N*-hydroxysuccinimide (NHS) is a reactive derivative of the dye interacting easily with amine groups. Although the toxicity profile of the Cy5.5 itself is unknown, the conjugated forms are widely studied as fluorescent labels,^16^ in biological monitoring^17^ and *in vitro* and *in vivo* imaging^18,19^ with little toxicity reported. The importance of multimodal contrast agents for bioimaging has been widely demonstrated in various studies.^20−23^

In the current work we present the synthesis of core–shell NPs with dual mode properties as contrast agents for optical and X-ray fluorescence bioimaging. The optimized synthesis of core–shell NPs on MoO_2_ core NPs was tailored and demonstrated as applicable to metallic Rh and Ru NPs with the same surface coating (PVP). Additionally, integration of the Cy5.5 fluorophore into the SiO_2_ coating of the core–shell NPs provides optical emission properties. The multimodal capabilities of the core–shell NPs were demonstrated *in vitro* with confocal microscopy, and *in situ* through small-animal multiplexed XFCT, showing the potential application of these NPs as contrast agents for both microscopic and macroscopic imaging.

## Results and Discussion

### Core Nanoparticles

Mo, Rh, and Ru based NPs were synthesized via hydrothermal and polyol methods. In both methods, the synthesis media, ethanol (EtOH) for Mo based NPs and ethylene glycol for Rh and Ru NPs, act as reducing agents in the reaction (Mo^6+^ to Mo^4+^, Rh^3+^ to Rh^0^, and Ru^3+^ to Ru^0^). The polymer, poly(vinlypyrrolidone) (PVP) has a complex role in the synthesis process, limiting the particle growth during the synthesis, capping, and stabilizing surface of the NPs formed.^24^

**Figure 1:**
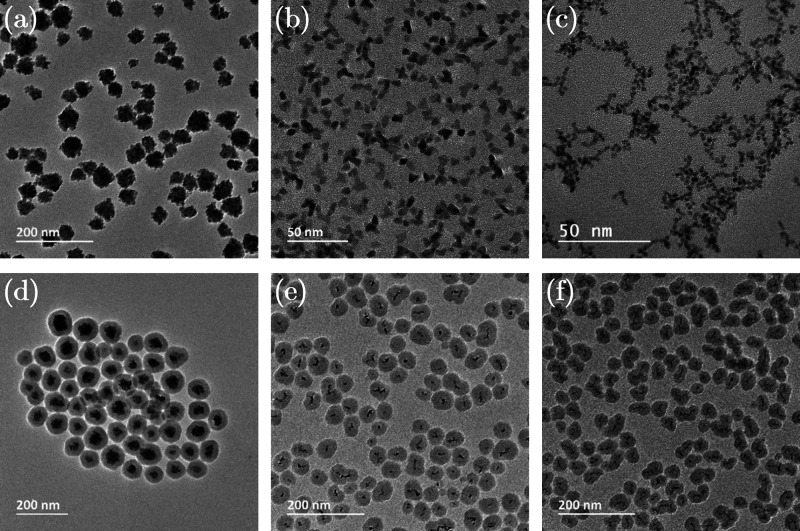
TEM micrographs of core and core–shell NPs: (a) MoO_2_, (b) Rh, (c) Ru, (d) MoO_2_–SiO_2_, (e) Rh–SiO_2_, and (f) Ru–SiO_2_ NPs.

The powder X-ray powder diffraction (pXRD) (Figure S1) pattern of the core NPs revealed the MoO_2_ phase as the dominant crystalline phase in the Mo-based NP (ICDD no.: 00-050-0739). Rh (ICDD card: 03-065-2866) and Ru (ICDD card: 01-089-4903) were obtained in metallic form, where the diffraction peaks are broadened due to the small crystallite size. Furthermore, the broad diffraction peak at ∼20° is ascribed to the presence of an amorphous coating layer.^25^ Surface charge and size distribution analyses performed on the NP samples are summarized in Table 1. MoO_2_ NPs exhibited a strong negative surface charge with a ζ-potential of −39 mV, demonstrating high colloidal stability. MoO_2_ NPs with a size of around 5 nm assembled in clusters with an overall average size of about 47 (±13) nm in the dry form (Transmission Electron Microscopy (TEM)), and 85 (±1) nm in the dispersed form (Dynamic Light Scattering (DLS)) (Table 1, Figure 1a). Rh NPs showed a predominantly spherical (minor triangular) morphology while Ru NPs were exclusively spherical. The TEM size was 6 (±1) nm and 2.4 (±0.4) nm, while the surface charges were +4 (±1) mV and 0 (±1) mV for Rh and Ru NPs, respectively. Despite the almost-neutral surface charge of these metallic NPs, they presented high colloidal stability most likely due to the steric effect induced by the PVP coating (Table 1, Figure 1 b, c). The presence of PVP on the surface of the core NPs was confirmed by Fourier Transform Infrared Spectroscopy (FT-IR) analysis (Figure S2), and the content was estimated by TGA (Figure S3).

**Table 1:** ζ-Potential, TEM size, DLS size and PDI for MoO_2_, MoO_2_–SiO_2_, Rh, Rh–SiO_2_, Ru, and Ru–SiO_2_ NPs

	ζ-Potential [mV]	TEM Size [nm]	DLS Size [nm]	PDI
MoO_2_	–39 ± 1	47 ± 13	72 ± 2	0.13
MoO_2_–SiO_2_	–51 ± 1	78 ± 12	150 ± 2	0.06
Rh	4 ± 1	6 ± 1	44 ± 1	0.26
Rh–SiO_2_	–48 ± 4	44 ± 8	101 ± 1	0.18
Ru	0 ± 1	2.4 ± 0.4	50 ± 1	0.19
Ru–SiO_2_	–47 ± 1	38 ± 8	94 ± 1	0.11

### SiO_2_ Coating on the Core Nanoparticles

MoO_2_ NPs were employed for tuning the SiO_2_ coating process, since they constitute the most promising candidate for XFCT due to the low background level and good signal-to-noise ratio (SNR).^7^ Details of the optimization process are presented in the Supporting Information. In particular, the present study focused on the optimization of the catalyst, Ethanolamine (EA), content (Figure S4) and NP concentration for the SiO_2_ coating process (Figure S5). The optimized parameters were further applied for SiO_2_ coating of Rh and Ru NP cores. Surface charge (ζ-potential) and size distribution analyses performed on the core and core–shell NPs are summarized in Table 1. The DLS size values were always significantly higher than the dry (TEM) size, revealing the contribution of adsorbed molecules on the NPs’ surface. The ζ-potential of the coated NPs proved the success of the coating process, accompanied by a decrease in surface charge from close to the isoelectric point (IEP) for core NPs to strongly negative values for the SiO_2_ coated NPs. The TEM micrographs of MoO_2_–SiO_2_, Rh–SiO_2_, and Ru–SiO_2_ NPs (Figure 1 d–f) showed a uniform SiO_2_ coating. The homogeneous SiO_2_ coating on the cores and the overall size of ∼100 nm make these NPs suitable for use in biomedical applications, and hence they were employed for further analyses.^26^

### Cy5.5-APTES Conjugation

**Figure 2:**
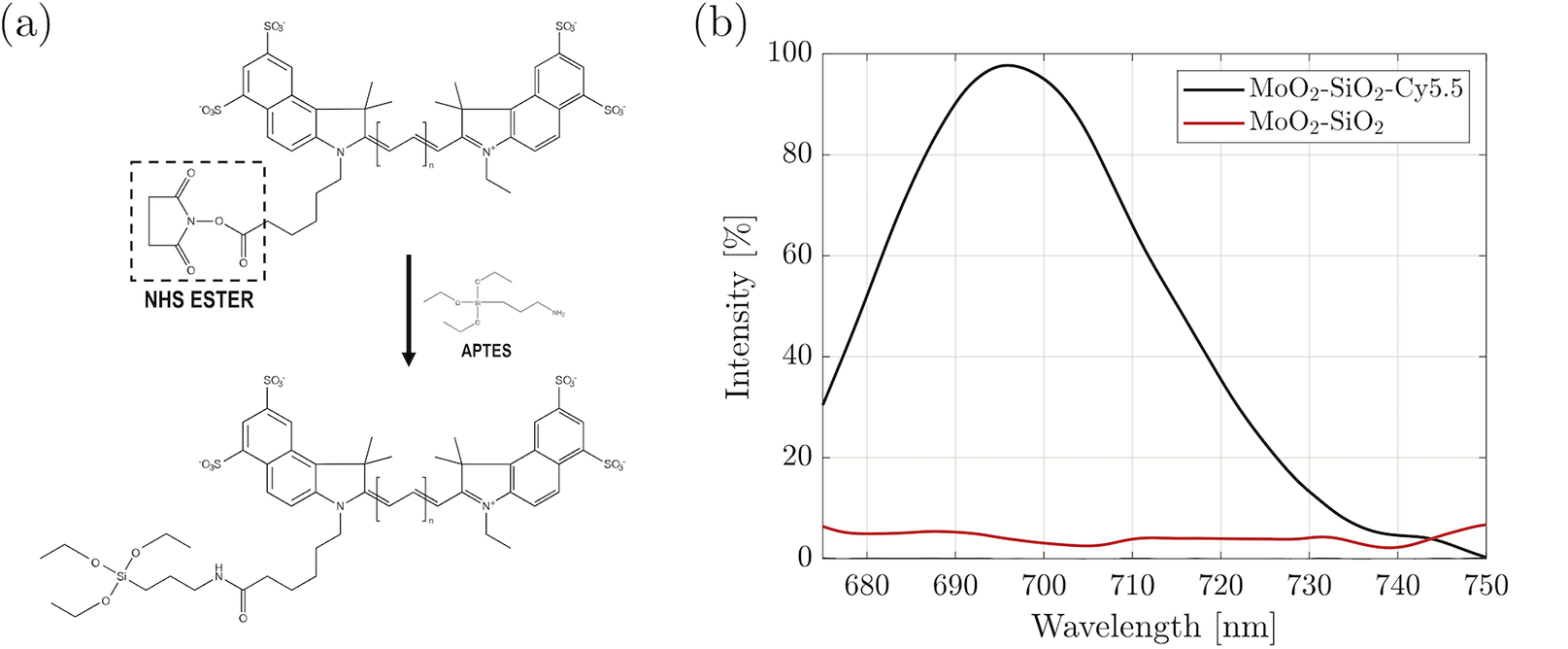
(a) Schematic representation of Cy5.5-NHS reaction with APTES, leading to the formation of the Cy5.5-APTES complex. (b) Emission spectra of MoO_2_–SiO_2_ core–shell NPs before and after conjugation with Cy5.5 (excitation at 665 nm).

For the XRF active core-SiO_2_ shell NPs to be used as dual mode contrast agents, the Cy5.5 dye was integrated into the SiO_2_ shell as a fluorophore. First, the Cy5.5 conjugation with (3-aminopropyl) triethoxysilane (APTES) was performed. The addition of APTES into the dispersion of Cy5.5-NHS in dimethylsulfoxide (DMSO) led to the conjugation reaction between the NHS ester group of the Cy5.5-NHS molecule and the carbon side chain of APTES, as schematically represented in Figure 2a. The absorption spectrum of Cy5.5-APTES revealed two peaks, at 630 and 675 nm. The optical fluorescence spectrum (excitation at 665 nm) exhibited a strong emission centered at 695 nm, characteristic of Cy5.5, revealing that the reaction process did not alter or quench the Cy5.5 molecule, as previously reported (Figure S6).^27^

To render the core–shell NPs with optical fluorescence, Cy5.5-APTES was conjugated to the SiO_2_ shell. The integration process is not just a physical entrapment of the dye into the pores of SiO_2_, but its covalent incorporation into the shell through condensation. Although dye conjugation did not alter the dry size and morphology of the core–shell NPs, the Cy5.5-conjugated NPs exhibited a slight increase, on the order of 10 nm, in the DLS size (Table S3, Figure S7). The change in the hydrodynamic size can be attributed to the presence of dye molecules on the surface of the SiO_2_ shell, increasing the hydrated size. Moreover, this is confirmed by the less negative surface charge for all the Cy5.5 conjugated core–shell NPs compared to the core–shell NPs without the dye, indicating the superficial Cy5.5-APTES partially lowers the magnitude of the charge provided by the SiO_2_-coating. This effect can be ascribed to the presence of positively charged amine groups of APTES on the surface of the NPs.^28^ Furthermore, the emission spectra of the Cy5.5-conjugated core–shell NPs confirmed that the fluorescence of Cy5.5 is preserved after embedding in the SiO_2_ matrix (Figure 2b). The emission spectrum was presented for MoO_2_–SiO_2_–Cy5.5 NPs, and similar spectra would be expected for Rh- and Ru-based Cy5.5 conjugated core–shell NPs. The emission peak at 695 nm for the doped Rh and Ru NPs highlighted the unaltered properties of Cy5.5 dye when embedded in the SiO_2_ shell, while no emission peak is detected for the core–shell NPs without Cy5.5 (data not shown). Furthermore, TEM and Scanning Electron Microscopy (SEM) imaging of the Cy5.5 conjugated core–shell NPs (Figure S7) underlined the reproducibility of the coating process resulting in spherical and uniform NPs without necking.

### Cytotoxicity Studies and Optical Fluorescence Microscopy

Prior to investigating the applicability of the synthesized particles in bioimaging as multimodal optical and X-ray fluorescence contrast agents, we assessed their toxicity profile on macrophages and investigated whether the SiO_2_ coating layer and the addition of Cy5.5 had any cytoprotective effect on the NPs. Macrophages are chosen as the model cell line, as they are the constituents of all body tissues with a major presence in organs with important barrier function from external offensives: lungs, liver, and spleen. Through phagocytosis, clearance, and secretion, the macrophages perform important roles in innate and adaptive defenses against external and internal aggressions with the ultimate role of restoring tissue homeostasis.^29^ The RAW 264.7 macrophage cells used in our study originate from BALB/c mice mononuclear cells transformed with Abelsson Leukemia virus. This cell line is a widely used model.^30−33^ NP toxicity on this macrophages cell line can give an indication of the potential toxicity *in vivo*. The cellular viability, as an indicator for the toxicity potency of an agent, can be affected in multiple ways. Consequently, our studies were focused on testing how the cellular activity is affected by the exposure to the NPs at different exposure times.

**Figure 3:**
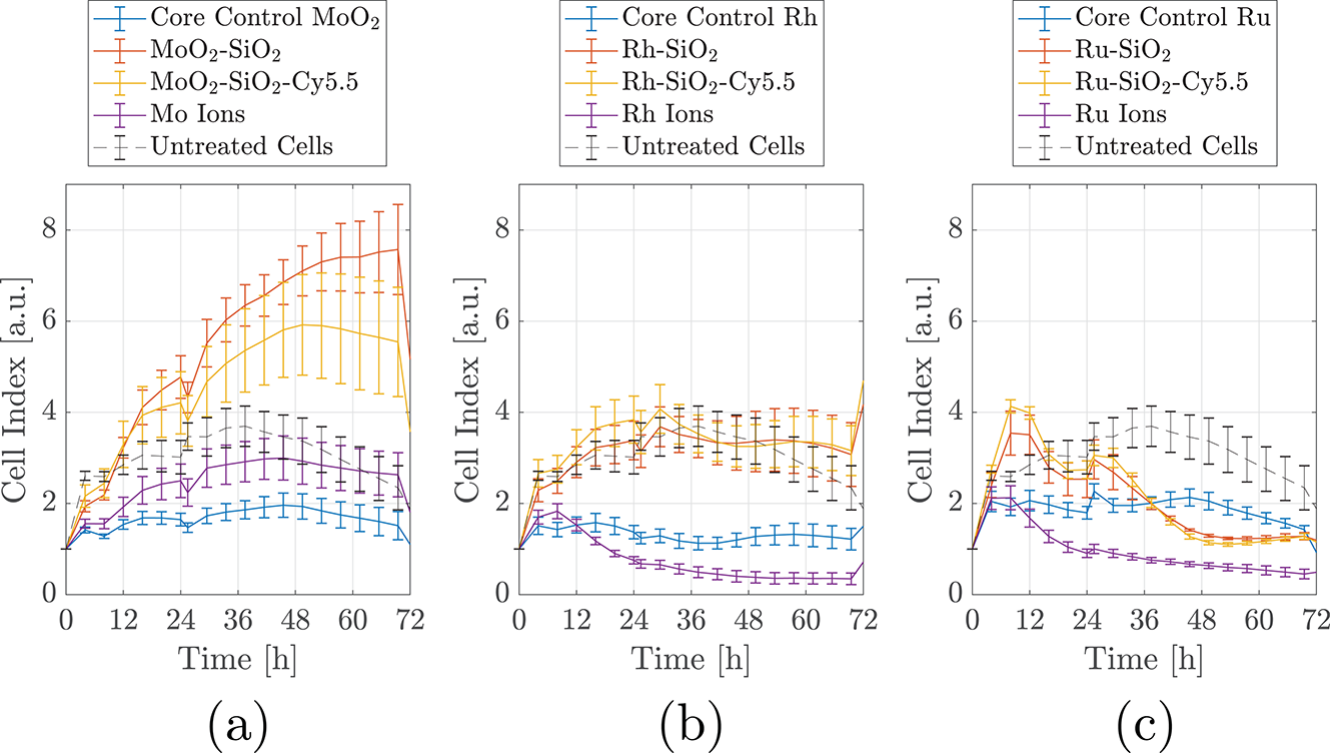
RTCA assay on RAW 264.7 cell lines with the three series of core and core–shell NPs, along with the free ionic forms of the three cores: MoO_2_ (a), Rh (b), and Ru (c). The cell index is normalized (CI = 1) at the time when NPs were added (*t* = 0).

We assessed the cytotoxicity of uncoated and SiO_2_-coated NPs and controls (core NPs, ions) in real time over a period of 72 h. Preliminary experiments performed with lower concentrations revealed no difference in toxicity between the uncoated and SiO_2_ coated NPs (data not shown). High core–shell NP concentrations (250 μg/mL) were chosen for the real-time cell analysis (RTCA) to ensure that cytotoxic discriminatory effects will be observed between uncoated and SiO_2_-coated NPs (Figure 3).

Mo, Rh, and Ru in ionic form impacted the viability and proliferation of RAW 264.7 cells differently. The CI curve of the cells exposed to Mo ions closely follows the CI curve of the untreated cells. When the cells are treated with Rh and Ru ions, their viability and proliferation CI curve was negatively impacted after 12 h. The negative trend of the CI curve could reflect either a toxicity effect of Ru ions or alternatively an interference with the electrical measurements. It is known that Rh- and Ru-based NPs are chemically inert and would not dissolve in physiological conditions.

Consequently, the potential toxic effect induced by Rh and Ru ions can be considered just hypothetical in biological settings.

The cells exposed to MoO_2_ NPs exhibited a similar trend to the Mo ions, with slightly lower CI. This can be due to the fact that although the concentration of Mo in the ionic solution and the MoO_2_ NPs suspension were identical, the toxic effect on cells of additional factors like crystallinity of the NPs cannot be ruled out.^28^ The metallic elements (Rh and Ru) were less toxic in their crystalline NP form proven by the higher cell viability and proliferation than when the cells were cultured in the presence of Rh and Ru in ionic form. This was expected, and it might be due to the chemically inert nature of these NPs in physiological conditions. To conclude, the behavior of the cells in the presence of MoO_2_, Rh, and Ru NPs was similar possibly due to the low concentration of the NPs the cells were exposed to.

No relevant differences were observed when the cells were incubated in the presence of core–shell NPs with or without Cy5.5, which reflects the nontoxicity of the fluorophore. MoO_2_–SiO_2_–Cy5.5 NPs displayed the highest CI curves when compared to the CI curves for the cells exposed to Rh–SiO_2_ and Ru–SiO_2_. It is known that RTCA measurements are influenced by the cell number and fluctuations in cell size and mass.^34,35^ At longer incubation times, a higher number of NPs will accumulate in the cells with the increase in the cells’ mass. The increase in CI observed for the cells incubated in the presence of MoO_2_–SiO_2_ NPs might be attributed consequently to an increment in the cells’ mass, besides the cell proliferation. As a note, MoO_2_–SiO_2_ NPs have a larger overall size when compared to Rh- and Ru-based core–shell NPs (Table 1), which indeed explains the higher CI for Mo-based core–shell NPs with respect to control, not observed for Rh- and Ru-based core–shell NPs.^36,37^ Ru-based core–shell NPs showed a reasonably high CI up to 24 h with a decline afterward. Understanding the causes of the observed differences in the toxicity of the core–shell NPs is the focus of ongoing research. Although the synthesized Ru NPs are used here as a proof of principle to show the multiplexing capability in XFCT, their application as contrast agents for *in vivo* imaging in their current form should be critically assessed, due to the observed *in vitro* cytotoxicity.

Furthermore, to qualitatively monitor the uptake process of the NPs by the macrophages, TEM analysis was performed, using a lower concentration of NPs to avoid interference from eventual toxicity. The micrographs (Figure S8) revealed that the uptake is time dependent with a visibly bigger accumulation of NPs in the cells at 24 h. The NPs were present in confined compartments in the cytoplasm, where they showed preserved morphology of the core–shell NPs.

We probed the viability of the macrophages exposed to the NPs with an end point assay at a time point in the RTCA time frame. The limited viability reduction in the presence of SiO_2_ coated NPs with and without Cy5.5 in the cytotoxicity assay was confirmed by live imaging (Figure S9), showing a small number of dead cells (≤4%) quantified in various field of views (FOVs). The presence of background signal in Figure S9 was ascribed to the emitted diffused light from the NPs, due to the absence of a pinhole in the live fluorescence microscope.

**Figure 4:**
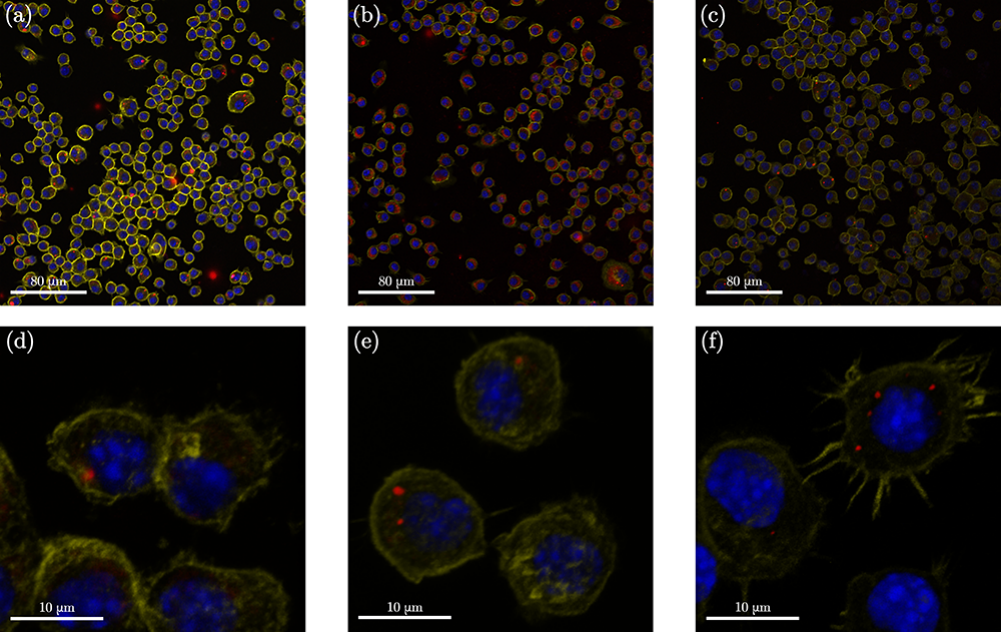
Confocal microscopy images of fixed and stained RAW264.7 Macrophages incubated for 24 h with the Cy5.5-conjugated (red) core–shell NP samples (250 μg/mL, in red) with three different cores, (a–d) MoO_2_, (b–e) Rh, and (c–f) Ru, at different magnifications (20× and 63×). DAPI (blue) and Alexa 555-Phalloidin (yellow) are markers for cell nuclei and actin filaments, respectively.

Cy5.5 conjugated NPs were tracked using confocal fluorescence microscopy. All sets of Cy5.5-conjugated NPs were readily detected as infrared signals (emission peak at 695 nm) in the cytoplasm of the macrophages exposed to the NPs for 24 h (Figure 4) and 72 h (Figure S10), confirming cellular uptake. At a higher magnification, the NPs were detected in distinct intracellular compartments (Supplementary File M1 (Confocal Imaging Movie); DAPI in blue, Alexa 488-Phalloidin in green and Rh–SiO_2_–Cy5.5 in red), corroborating the TEM observations. No signals were detected in the infrared range (Figure S11) from the cells incubated with the core–shell NPs in the absence of Cy5.5 imaged at 24 and 72 h incubation times. To additionally investigate whether the NPs accumulated in lysosomes as a part of the natural phagocytic cell pathway, the cells were incubated with Lysotracker.

**Figure 5:**
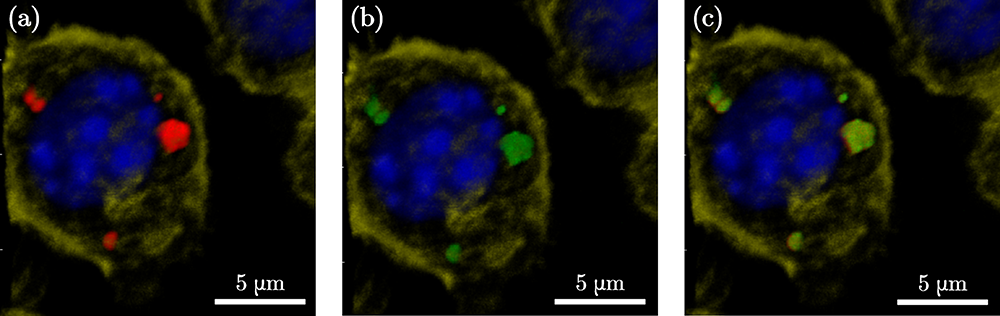
Confocal microscopy images of fixed and stained RAW Macrophages incubated for 72 h with MoO_2_–SiO_2_–Cy5.5 NPs (250 μg/mL, red), (a–c). DAPI (blue) and Alexa 555-Phalloidin (yellow) are markers for cell nuclei and actin filaments, respectively. Lysotracker (green) is a marker for lysosomes (b and c).

The overlap of Cy5.5 signal (red) with Lysotracker signal (green) demonstrated that Cy5.5-conjugated MoO_2_–SiO_2_ NPs were phagocytized and localized inside cells, into the lysosomal compartments at 72 h (Figure 5). The presence of the Cy5.5 conjugated NPs in the cells was also observed in the dividing cells, indicating that cell division was unaffected by their presence (Figure S12). The confocal microscopy results reveal doped core–shell NPs’ potential for intracellular tracking and localization. For future *in vivo* settings, the doped NPs can thus be localized in tissues and in specific cellular environments, *via* histological analysis in addition to the whole-body optical fluorescence tracking.

### X-ray Fluorescence Computed Tomography Performance

**Figure 6:**
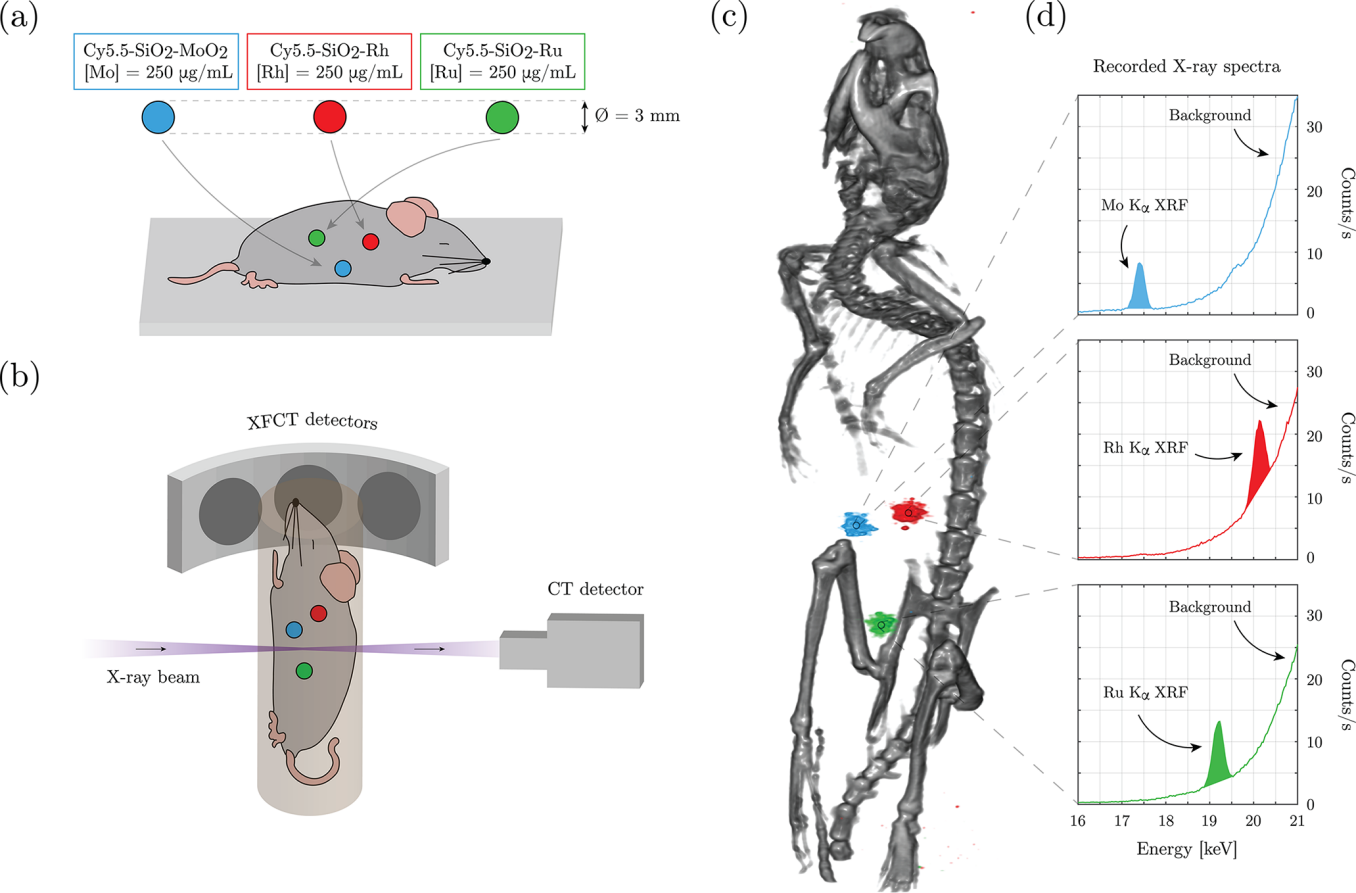
XFCT *in situ* experiment on a sacrificed mouse, where spherical sample holders containing the core–shell NPs, based on Mo, Rh, and Ru, were inserted in the abdominal region (a); the imaging arrangement (b); 3D visualization of reconstructed tomographic data sets demonstrating multiplexed imaging of the different core elements of the NPs (c); XFCT detector spectra recorded for 5 min at the position of the three sample holders showing the Kα XRF peak for the three elements together with the increasing Compton scattering background at higher energies (d).

An XFCT *in situ* experiment was performed on a sacrificed mouse, with spherical sample holders containing the contrast agents inserted in the abdominal region (Figure 6a). The imaging arrangement is displayed in Figure 6b. XFCT for whole-body multiplexed imaging of the synthesized NPs is demonstrated in Figure 6c and d and Supplementary File M2 (*in situ* XFCT). Figure 6c shows the tomographic reconstruction of the XFCT data (color) overlaid on the CT data (grayscale). The three spherical sample holders could be clearly distinguished from the background in the XFCT reconstruction. Moreover, the Kα XRF signal from the different set of NPs of respective core elements (Figure 6d, Mo: 17.45 keV, Ru: 19.28 keV, Rh: 20.22 keV) could be spectrally separated with no overlap. Background signal, arising from Compton scattering of the ∼24 keV incident X-ray photons, increased for higher energies up to a maximum of ∼23 keV. The background contribution at each XRF peak was estimated and subtracted in the XFCT reconstruction workflow, explaining why the background was not visible in the 3D visualization. Nevertheless, the effect of the background contribution could be seen in the raw 2D projections (Figure S13) where the best signal-to-background ratio (SBR) is found for MoO_2_–SiO_2_–Cy5.5 NPs. As higher background contribution at similar signal levels negatively affects sensitivity—i.e., lower SBR is associated with a lower sensitivity—it was expected that the MoO_2_–SiO_2_–Cy5.5 NPs had a lower minimum detectable concentration than Rh–SiO_2_–Cy5.5. We note that the SiO_2_ coating layer did not contribute to any significant self-absorption of XRF, as the quantitative reconstruction algorithms confirmed within ±10% accuracy the core concentration obtained from Inductively Coupled Plasma-Optical Emission Spectrometry (ICP-OES), as expected from the previous *in vivo* studies on the core NPs.^5,25^ Furthermore, simultaneous detection of the signal from the three core NPs with one single scan clearly demonstrated the multiplexing capability of the technique, demonstrating its potential use for multitargeting XFCT *in vivo* applications.^38^ It is important to note that no claims are made for correlative imaging between XFCT and optical fluorescence imaging at this stage, but they rather constitute complementary imaging techniques.

Different concentrations of NPs for *in vitro* and *in situ* investigations were deliberately chosen, in order to highlight their multiple potentialities. With a further reduction of the SiO_2_ shell thickness, the concentration variations between the uncoated and SiO_2_ coated NPs will be minimized, making the utilized dose more tunable for *in vivo* bioimaging applications.

## Conclusions

In this work we presented a synthetic strategy and validation of dual modality optical and X-ray fluorescent NP platforms for bioimaging, and demonstrated their efficiency through confocal microscopy and XFCT evaluations. The cores were selected from previously established potential XFCT contrast agents, while the SiO_2_ coating increased their biocompatibility, offering a platform for fluorophore conjugation with Cy5.5, and consequently adding on the functionality of the NPs. The as-synthesized core–shell NPs exhibited uniform spherical morphology and strong negative charge ensuring high dispersibility in neutral pH of the biological media. Different concentrations of NPs were chosen for *in vitro* and *in situ* characterizations to match the employed techniques and highlight their multiple potentialities. The decreased cytotoxicity of the SiO_2_ coated NPs compared to the corresponding core NPs and the confocal intracellular detection and localization was demonstrated. Furthermore, this study proved the possibility to localize few-mm sized accumulations of the contrast agents in a whole-body small-animal setting by detecting the corresponding X-ray fluorescence signals, thus making possible the detection of few-mm early stage tumors, supported by previously proven imaging sensitivity *in vivo* down to 50 μg/mL,^6^ which is within the range of observed passive NP dose in tumors.^5^ Finally, the multiplexed XRF detection of the three NPs combined with the possibility of additional real time detection of the conjugated optical probe could lead to the identification of multiple targets simultaneously, potentially representing an expanded diagnostic toolbox. Future studies, in particular *in vivo* imaging studies of targeted NPs, are warranted to establish the potential utility of the developed contrast agents for preclinical targeting and bioimaging studies.

## Methods

### Materials

Rhodium(III) chloride hydrate (RhCl_3_·*x*H_2_O, Rh 38.5%–45.5%), Ruthenium(III) chloride hydrate (RuCl_3_·*x*H_2_O, Ru 38%–40%), Ethylene glycol (EG, >99%), Poly(vinyl-pyrrolidone) (PVP, 55 kDa), Ammonium heptamolybdate (AHM, (NH_4_)_6_Mo_7_O_24_·4H_2_O), (3-Aminopropyl)triethoxysilane (APTES, H_2_N(CH_2_)_3_–Si(OC_2_H_5_)_3_), Cy5.5 Mono NHS Ester (Cy5.5-NHS), Triethylamine (TEA, (C_2_H_5_)_3_N, ≥99%), Dimethyl sulfoxide (DMSO, (CH_3_)_2_SO), Hydrochloric acid (HCl, 37%), Tetraethyl orthosilicate (TEOS, Si(OC_2_H_5_)_4_, ≥ 99%), Ethanolamine (EA, NH_2_CH_2_CH_2_OH, ≥99%), Dulbecco’s modified Eagle medium (DMEM), Fetal Bovine Serum (FBS), and Murine macrophages (RAW 264.7, 91062702-1VL) were all purchased from Sigma-Aldrich. Ethanol (EtOH, CH_3_CH_2_OH, 99.7%) was bought from Solveco. All fluorescent probes, NucGreen Dead 488 ReadyProbes Reagent (SYTOX Green), 4′,6-diamidino-2-phenylindole (DAPI), NucBlue Live reagent (Hoechst 33342 dye), LysoTracker Green DND-26, Alexa Fluor 555 Phalloidin, and Alexa Fluor 488 Phalloidin were all purchased from ThermoFisher.

### Synthesis of Core Nanoparticles

MoO_2_, Rh, and Ru NPs were synthesized as described in detail elsewhere.^5,25,39^ Briefly, MoO_2_ NPs were synthesized *via* a hydrothermal method. In a typical process, 3.6 mM AHM was dissolved in 54 mL of deionized (DI) water and 24 mL of EtOH, followed by the addition of 0.29 mM PVP, and stirring for 30 min. After PVP is completely dissolved, the transparent solution was transferred to a stainless-steel autoclave with Teflon lining, and the synthesis reaction was performed at 180 °C for 18 h. After the synthesis, the dark particle suspension was collected and washed by successive centrifuging and redispersion in DI water.

The Rh and Ru NPs were synthesized through a polyol reduction method.^25^ 0.2 mmol of Rh precursor and 4 mmol of PVP were mixed in 20 mL of EG, and the dispersion was heated to the nucleation temperature 80 °C for 15 min, under continuous stirring. Then, the temperature was set to 115 °C (focusing temperature) and maintained for 1.5 h. Similarly, Ru NPs were synthesized by nucleation at 140 °C, with the reaction continuing at 150 °C for 1.5 h. The as-synthesized NPs were then washed three times with acetone by alternated dispersion and centrifuging cycles. Finally, the collected NPs were dispersed in DI water.

### Cy5.5-APTES Solution

For the conjugation of Cy5.5 with APTES, 1 mg of Cy5.5-NHS was dispersed in 50 μL of DMSO. While the mixture was stirring, 0.3 μL APTES was added, followed by the addition of 0.2 μL of TEA. The mixture was then stirred for 24 h at room temperature in a dark environment. The as-formed Cy5.5-APTES solution was then stored at 4 °C.^27^ For optical absorption and fluorescence characterization of the complex, 2 μL of Cy5.5-APTES solution were dispersed in 1 mL of DI water prior to measurements.

### SiO_2_ Shell Formation

The SiO_2_ coating on the core NPs was performed by a modified sol–gel method by tuning several reaction parameters.^40^ Typically, a solution of 3.75:1 molar ratio of ethanol/water was prepared. Core NPs were then added under stirring, together with 0.01 M TEOS, resulting in final concentrations of 150 μg/mL for MoO_2_ and 50 μg/mL for Rh and Ru NPs. After 30 min, 0.16 M EA was slowly added in the suspension and reacted for 2 h. The Cy5.5 conjugation was accomplished by adding Cy5.5-APTES solution (4 μL), corresponding to 5.5 μM in a final reaction volume of 19 mL, 1 h after the addition of EA. The obtained core–shell NPs were then washed by centrifuging and dispersion in EtOH, and subsequent redispersion in DI water.

### Characterization Techniques

The surface charges (ζ-potentials) and hydrodynamic (DLS) sizes were measured in triplicates on diluted solutions at neutral pH using the Zetasizer Nano ZS90 system (Malvern, UK). Reported DLS size values are volume-average values. TEM (JEM-2100F, 200 kV, JEOL) was employed to evaluate the morphology and size of dried NPs. Copper grids were used, where 40 μL of the samples were drop-casted and dried at room temperature. For the TEM size analysis, at least 350 NPs/clusters in different field of views were measured. SEM (FEI Nova 200) provided further characteristics of NP morphology, including an overview of the samples. Dried samples were prepared on a graphite-coated aluminum holder; several acceleration voltages were used for imaging, ranging from 10 to 20 keV. Ultraviolet–Visible Spectrophotometry (UV–vis, NP80, Implen) and PL (Spectrofluorometer, FP-8300, Jasco) were used for the analysis of the Cy5.5-APTES complex as well as for the confirmation of the Cy5.5 integration in the SiO_2_ shell of the core–shell NPs. For PL the excitation and emission bandwidth were 5 nm, the scan speed was 100 nm/min, and the excitation wavelength was 665 nm. The crystallographic phase of the core NPs was determined using XRPD (Panalytical Xpert Pro alpha powder, PANalytical) with Cu Kα radiation, a 1.5406 Å wavelength, and a scanning rate of 0.13° min^–1^. The presence of the PVP on the surface of the core NPs is confirmed by FT-IR (Thermo Fisher Scientific). The quantification of PVP adsorbed on the surface of the NPs was done by TGA (TGA550, TA Instruments). ICP-OES (iCAP 6000 series, Thermo Scientific) was employed to determine the concentration of metallic species in the NP stock solutions.

### Limulus Amebocyte Lysate (LAL) Assay

Before proceeding with *in vitro* analysis, the NP suspensions were tested for lipopolysaccharides (LPS) contamination.^41^ The LAL assay Endosafe-PTS (Charles River) was applied to the stock NP suspensions and the sterile DI water used for preparing the final stock suspensions. The test was carried out following the manufacturer’s instructions using PTS cartridges with a sensitivity of 0.005 EU/mL. All the stocks and the DIW had LPS values below the maximum admissible limit of 0.1 EU/mL.^42^

### *In Vitro* Toxicity Studies on Macrophages

Toxicity tests were performed on the murine macrophage cell-line (RAW 264.7, 91062702-1VL, Sigma-Aldrich). NP concentrations that the cells were exposed to were expressed as total weight, in μg/mL, for the NPs’ core and as the overall weight of the core and the SiO_2_ shell for the core–shell entities. In all the tests, the ionic and NPs form of Mo, Rh, and Ru in concentrations of 90 μg/mL, 16 μg/mL and, correspondingly, 27 μg/mL were used (estimated core concentrations in the core–shell NPs, *via* ICP-OES). The ionic solutions were used as extra controls to address the toxicity induced solely by the ions (in the eventuality of dissolution).

Cell viability and proliferation were determined in real time using an automated cell analyzer (xCELLigence Agilent, St Clara USA) that measures electrical impedance obtained as the result of confluence in a monolayer cell culture, referred to as the cell index (CI). Impedance increases in proportion to the number and/or size of the adherent cells. Approximately 7000 cells/well were plated in RTCA plates, in quadruplicates per condition. The cells were allowed to adhere to the plate surface for 24 h before adding the NPs (time = 0) and were followed for 72 h. Untreated cells were used as proliferating controls, and the ionic solutions at concentrations equimolar to the core NPs were used as additional controls.

### *In Vitro* TEM Studies

The RAW 264.7 macrophages were seeded in 12 well plates (Sarstedt), 24 h before NP exposure (200 000 cells/well), and exposed to NPs with a concentration of 50 μg/mL for 2 or 24 h. After incubation, the cells were washed three times with PBS, detached with TrypLe (Invitrogen), pelleted, and fixed with 0.9% NaCl solution containing 2.5% glutaraldehyde. The pellets were postfixed in 2% osmium tetroxide in 0.1 M phosphate buffer, pH 7.4 at 4 °C for 2 h, dehydrated in ethanol followed by acetone, and embedded in LX-112 (Ladd, Burlington, Vermont, USA). Ultrathin sections (∼70 nm) were cut using a Leica Ultracut EM UC6 (Leica, Wien, Austria). The sections were contrasted with uranyl acetate followed by lead citrate and examined by SEM (Quanta 650) with a STEM II detector using 30 kV acceleration voltage (Thermo Fisher).

### Live Imaging and Confocal Microscopy

For intracellular localization of the engulfed NPs, RAW 264.7 murine macrophages were plated in chamber slides at concentrations of 20 000 cells/well and incubated overnight to form a monolayer. Thereafter, the cells were exposed for 24 h to the core–shell NPs, with and without Cy.5.5 conjugation, dispersed in cell growth medium at a concentration of 250 μg/mL. Subsequently NPs were removed, and the cells were washed two times with PBS and incubated with NucGreen Dead 488 ReadyProbes Reagent (SYTOX Green) and with NucBlue Live reagent (Hoechst 33342 dye). Live images of the cells were obtained using an EVOS 5000 Imaging System (Thermofisher Scientific, Ma, USA).

For the confocal imaging, cells were exposed to the core–shell NPs without and with Cy5.5 conjugation (250 μg/mL) for 24 or 72 h. After NP exposure, the cells were fixed in 4.5% buffered paraformaldehyde for 10 min, permeabilized with 0.1% Triton X100 for 15 min, blocked with 3% BSA, and incubated with 4′,6-diamidino-2-phenylindole (DAPI), Alexa Fluor 555 Phalloidin (or Alexa Fluor 488 Phalloidin), and Lysotracker Green DND-26 for staining nuclei, actin filaments, and acidic intracellular compartments (lysosomes), respectively. Confocal Images were obtained using a laser microscope LSM700 (Zeiss, Overkochen Germany), with 405, 488, 555, and 639 nm (for Cy5.5) laser lines. Two objectives were employed, 20× air/dry (for wide view) and 63× oil (for detailed view and NPs’ intracellular localization).

### *In Situ* Small-Animal XFCT

To investigate the potential of the synthesized NPs to be used as contrast agents for whole-body imaging using X-ray fluorescence, an experiment was designed to simulate a small-animal XFCT setting. Three spherical sample holders with 3 mm inner diameters were filled with the dispersion of MoO_2_–SiO_2_–Cy5.5, Rh-SiO_2_–Cy5.5, and Ru-SiO_2_–Cy5.5 NPs, respectively. The concentration of the dispersions was fixed at 250 μg/mL of the core elements (Mo, Rh, and Ru), which generate the XRF signal. The concentration was chosen within previously observed local organ doses,^6^ and the size of the sample holders were small enough to be similar to biomedically interesting features (e.g., small tumors). Subsequently, the spherical sample holders were surgically inserted in the abdominal region (5–10 mm depth) of a mouse previously sacrificed for an unrelated study. At 10 mm depth, the estimated XRF tissue transmission is 31%, 44%, and 40%, respectively, for Mo, Rh, and Ru, which is more than sufficient for the whole-body small-animal XFCT. The mouse was then positioned in an in-house dual-modality XFCT and CT imaging arrangement.^6^ The spectral resolution of the XFCT imaging arrangement enables multiplexed imaging of the three different core materials (Mo, Ru, and Rh).^7^

Thirty projection images were acquired over 180° for dual XFCT and CT imaging. Each projection image was acquired with a step size of 200 μm and exposure time of 10 ms per step, resulting in a total acquisition time of ∼1.5 min for each axial slice (with 200 μm thickness). Depending on the time constraints, the number of slices to be imaged can be selected to be within a region of interest. The described settings have been demonstrated to be suitable for *in vivo* imaging, with an estimated radiation dose of ∼25 mGy for a liver-region 1-h tomographic scan.^6^ CT data were later reconstructed using a standard filtered back projection algorithm, while the XFCT data were reconstructed using an in-house developed quantitative iterative algorithm. Following the tomographic acquisition, each sample holder was located in the 2D projections and a stationary XRF spectrum was recorded for 5 min at their respective locations. More details on the imaging arrangement, including X-ray source, optics, and detector characteristics, can be found in a previous study.^6^

## Supporting Information Available

Detailed characterization of core NPs, optimization of SiO_2_ coating procedures, Cy5.5 conjugation, and additional analyses and results for in vitro and in vivo experimental techniques. (PDF)
Confocal imaging movie: Z-Stack at 63× detecting NPs in distinct intracellular compartments, showing DAPI in blue, Alexa 488–Phalloidin in green, and Rh-SiO_2_-Cy5.5 in red. (MP4)
*In situ* XFCT: animation of the tomographic reconstruction of the XFCT data (color) overlaid on the CT data (grayscale). In blue, red, and green, the signal detected from the contrast agents, respectively, molybdenum, rhodium, and ruthenium. (MP4)
