# Supplementary material for: Optical and X-ray Fluorescent Nanoparticles for Dual Mode Bioimaging
Source: ACS Nano. 2021 Feb 15;15(3):5077–85. doi: 10.1021/acsnano.0c10127 (PMC8028327; doi:10.1021/acsnano.0c10127)
Supplement: Supplementary file 1 — nn0c10127_si_001.pdf [file nn0c10127_si_001.pdf]

Supporting Information

Optical and X-Ray Fluorescent Nanoparticles for Dual Mode Bioimaging

Giovanni M. Saladino*, Carmen Vogt, Yuyang Li, Kian Shaker, Bertha Brodin, Martin Svenda, Hans M. Hertz and Muhammet S. Toprak*

Department of Applied Physics, Biomedical and X-Ray Physics,
KTH – Royal Institute of Technology, SE 10691 Stockholm, Sweden
Corresponding authors: saladino@kth.se (GMS); toprak@kth.se (MST)

Characterization of Core Nanoparticles

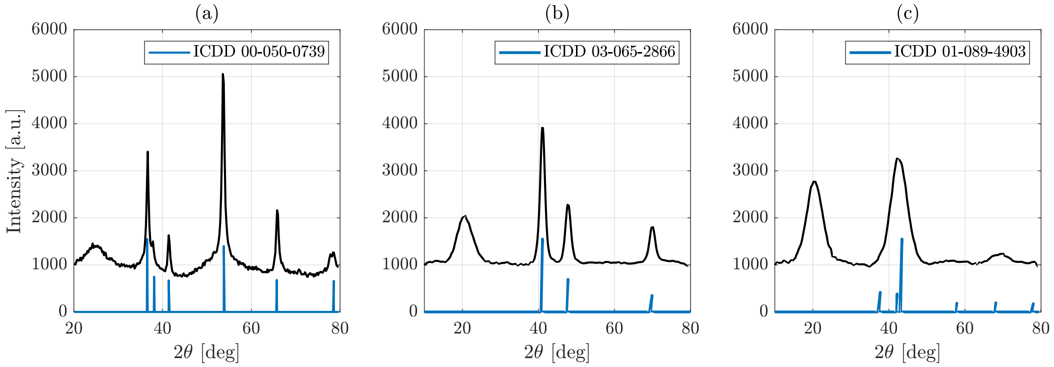

Figure S1. Powder X-ray diffraction (pXRD) patterns of PVP-coated core NPs: (**a**) MoO_2, (**b**) Rh and (**c**) Ru NPs. The corresponding matching ICDD card numbers are indicated for each sample on the XRPD pattern.

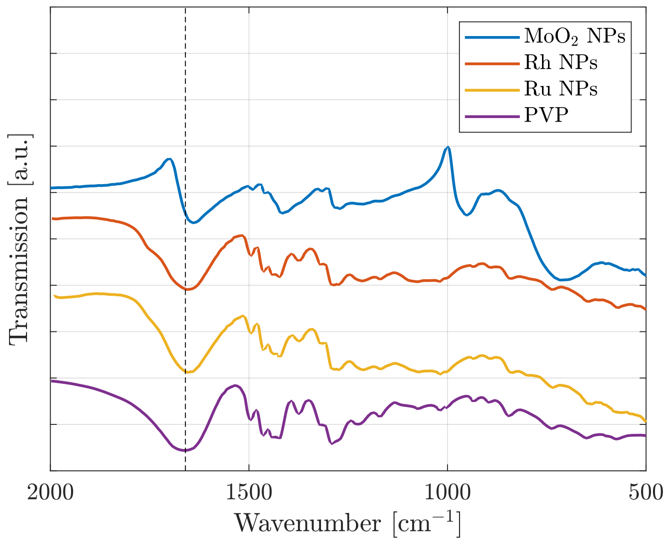

Figure S2. FT-IR spectra of pure PVP and core NPs coated with PVP.

The chain length of 55 kDa PVP was estimated as 88 nm.[1] By comparing the hydrodynamic sizes reported in Table 1, the circumference of ideal spherical NPs with the given hydrodynamic sizes (72 nm for MoO_2, 44 nm for Rh and 50 nm for Ru) cannot be wrapped around by a single stretched PVP chain. It would require theoretically at least three stretched PVP chains for MoO_2 and two chains for Rh and Ru NP agglomerates to be surrounded in a planar configuration. Though, the given chain length of around 90 nm is for a PVP molecule in free movement in aqueous liquid and might change upon conjugation to surfaces. The density attachment on the NPs surfaces might additionally induce various conformational modifications. The presence of a large amount of PVP around Rh and Ru NPs as compared to MoO_2 NPs (TGA data in Supporting Information) can be attributed to the larger surface area of Rh and Ru NPs for the same weight that is available for PVP attachment. The presence of larger amount of PVP may also suggest the occurrence of multiple layers of PVP around Rh and Ru NPs, resulting in larger sizes identified by the DLS measurements compared to their dried size (TEM).

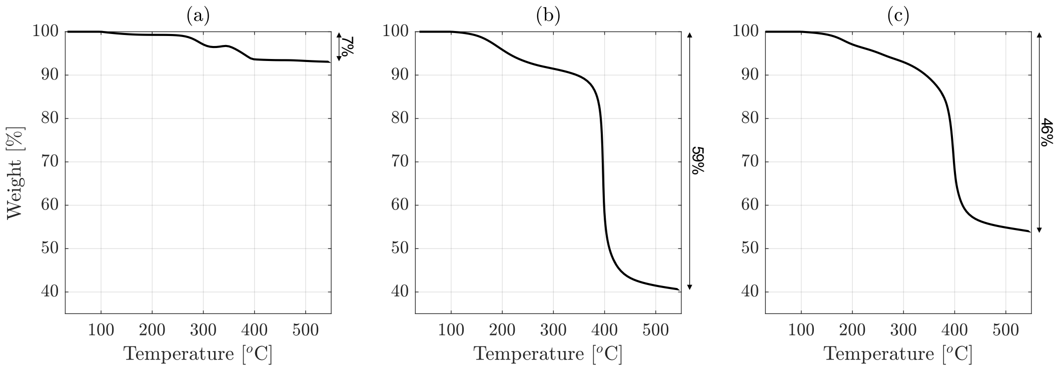

Figure S3. Thermogravimetric analysis of PVP capped NPs. The weight loss from 110 to 550 °C is ascribed to the decomposition of PVP on the surface of the (**a**) MoO_2, (**b**) Rh and (**c**) Ru NPs.

Optimization of SiO_2 Coating on the Core Nanoparticles

For the formation of SiO_2, or core-shell architectures with SiO_2 shell, a catalyst that creates a basic pH is required.[2] Traditionally, ammonia is the most commonly used catalyst promoting the SiO_2 formation by increasing the pH, *i.e.* OH^- ions concentration. In addition to the catalytic role, the increase in OH^- ion concentration increases the ionic strength of the media with a positive role in increasing the size of the final NPs.[3,4]

MoO_2 (Mo^{4+}) in media with high pH and/or high ionic strength can undergo oxidation to MoO_3 (Mo^{6+}) that has a higher solubility in water.[5–8] This process of oxidation and dissolution is accelerated for MoO_2 in NP form due to their high surface area/volume ratio. Consequently, to obtain core shell architectures with MoO_2 as the cores and SiO_2 as the shell, alternative methods with catalysts that work at lower pH and/or induce a minimal increase in ionic strength of the reaction media are desirable. Ethanolamine (EA) is an ideal candidate as a catalyst for the formation of SiO_2 shell as it induces minimum increase in the ionic strength of the reaction media, and operation at lower pH (around 7).[9] Moreover, it has a stronger catalytic effect than ammonia at the same concentration, increasing the rate of the reaction.[10] To the best of our knowledge, this is the first report of using EA as the catalyst in the formation of NPs with a SiO_2 shell.

The first set of experiments aimed at finding an optimal concentration of EA to promote the hydrolysis and condensation of the SiO_2 precursor (TEOS). Three different concentrations of EA were used, as summarized in Table S1, while keeping constant the NP concentration (50 µg/mL in the EtOH:water solution). The ζ-potential measurements revealed a significant increase of the (absolute value of the) surface charge for the core-shell NPs (-46 mV) synthesized using 0.08 M and 0.16 M EA compared to the surface charge of the core NPs (-39 mV). For the highest concentration of EA (0.24 M) the surface charge decreased to lower absolute values (-40 mV). This variation in ζ potential measured at the same pH for differently sized core-shell NPs is in agreement with the earlier observations, whereas a decrease in surface charge with increasing diameter of SiO_2 NPs was observed.[11] The hydrodynamic size increased with increasing EA concentration, reaching about > 2 times larger values as compared to the average size of the core NPs (0 M; no TEOS or EA). This systematic increase in the average dispersed size of the final core-shell NPs observed with increasing EA content was due to the increased kinetics of SiO_2 condensation reaction, as it led to consumption of larger quantity of TEOS precursor in the same reaction time. These results are also in line with the earlier reports on increased final size of SiO_2 NPs with the increase in EA concentration.[10] The TEM analysis (figure S4) confirmed that the increase in the size of core-shell NPs with

the increase in catalyst (EA) concentration is due to the increased SiO_2 shell thickness. Furthermore, for 0.08 M EA, free SiO_2 NPs are present in all fields of view probably because SiO_2 does not exclusively condense on the surface of the core NPs. This observation is also in agreement with the slightly higher PDI (an indicator of size distribution in the sample, where a value less than 0.3 relates to a homogeneous population) in DLS. Shell formation and growth around the core NPs is a continuous process of SiO_2 nuclei formation and their aggregation on the surface of the core NPs. This is known as heterogenous nucleation, where the concentration of SiO_2 precursor (in relation to the number concentration, or total surface area of core NPs) and the concentration of catalyst plays a significant role. An unbalance of the conditions may lead to homogenous nucleation of SiO_2, resulting in free standing, secondary SiO_2 NPs particles as in the studied case. When the EA concentration was increased this problem was avoided, revealing the significant role of concentration ratio between the precursors used.[4]

The NPs obtained using 0.16 M EA have a spherical morphology, with almost exclusively single cluster core-SiO_2 shell architecture and no free SiO_2 NPs formation. When higher concentrations of EA (0.24 M) were used, a partial dissolution of the MoO_2 cores is present (figure S4). This fact is possibly explained by a more porous structure of the formed SiO_2 shell induced by a 50% higher concentration of the catalyst.[10,12] The increased shell porosity results in a greater water access to the core and its consequent partial oxidation and dissolution. Consequently, for the next steps in the optimization process, the optimal concentration of EA was chosen as 0.16 M.

Table S1. ζ-Potential, TEM size, DLS size and PDI for MoO_2-SiO_2 core-shell NPs, using different concentrations of EA, while keeping constant the concentration of other precursors.

**Ethanolamine (EA)**	**0.08 M**	**0.16 M**	**0.24 M**
ζ-Potential [mV]	-47 ± 2	-46 ± 3	-40 ± 1
TEM Size [nm]	81 ± 12	109 ± 13	113 ± 13
DLS Size [nm]	159 ± 1	164 ± 3	178 ± 2
PDI	0.09	0.06	0.06

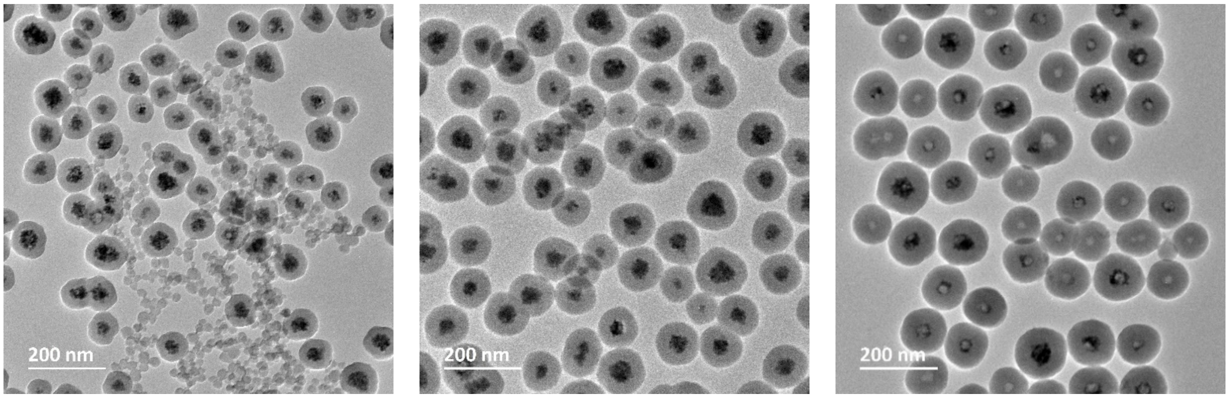

(a) **(b)** **(c)**

Figure S4. TEM micrographs of MoO_2-SiO_2 core-shell NPs, using three different concentration of EA. (**a**) EA 0.08 M, (**b**) 0.16 M and (**c**) 0.24 M. Water, EtOH, and TEOS concentrations were kept constant.

The second set of experiments was performed to tune the thickness of the SiO_2 shell without altering the spherical morphology of the resultant core-shell NPs or inducing agglomerations. The SiO_2 coating of NPs is well-known for low sample yield, mainly due to the use of diluted core NP dispersions in order to

have a better control of the process. As the aimed application is XFCT-related cellular imaging, an increase in the synthesis yield is desirable, in order to obtain high concentrations of the stock core-shell NP suspensions. We tuned the SiO_2 shell thickness by increasing the concentration of the MoO_2 NPs, leading to an increase in the number of the core cluster NPs, *i.e.* higher surface area for the condensation of SiO_2, while keeping the same concentration of the SiO_2 precursor and catalyst. Four concentrations of MoO_2 NPs were tested (Table S2). An increase in NPs concentration from 50 µg/mL to 200 µg/mL MoO_2 led to a thinner SiO_2 coating as the total surface area of NPs to be covered grew to 4 times larger for 200 µg/mL compared to 50 µg/mL MoO_2 NPs. This was confirmed by both DLS and TEM size measurements, where a significant size decrease was observed when increasing the MoO_2 NPs concentration from 50 µg/mL to 200 µg/mL. While PDI was almost constant for samples from 50 µg/mL to 150 µg/mL core concentrations, a non-negligible increase in the PDI was observed for 200 µg/mL MoO_2 NPs concentration, indicating a possible agglomeration of the formed NPs at this core concentration. The ζ-potential values for the core-shell NPs in this series were very similar. The decrease in the overall size of the SiO_2 coated NPs with the increase in concentration of the core NPs was confirmed by TEM (figure S4b and figure S5) with the overall size of the core-shell NPs decreasing from ~110 nm for 50 µg/mL to ~75 nm for 200 µg/mL. Furthermore, a slight increase in the PDI is observed, when using 200 µg/mL MoO_2 NPs concentration. The concentration of 150 µg/mL MoO_2 core NPs was the one that resulted in core-shell NPs with a thin SiO_2 shell, retaining the overall spherical shape and keeping the agglomeration to minimum (PDI under 0.1) with the dry size under 100 nm (TEM) and hydrodynamic diameter of 150 nm (DLS size). In the light of these findings, the concentration of MoO_2 NPs was fixed at 150 µg/mL, for the subsequent steps.

Table S2. ζ-Potential, TEM size, DLS size and PDI for MoO_2 - SiO_2 core-shell NPs, using four different concentrations of MoO_2 NPs, while keeping constant the concentration of other precursors.

**MoO_2 Concentration**	**50 µg/mL**	**100 µg/mL**	**150 µg/mL**	**200 µg/mL**
ζ-Potential [mV]	-46 ± 3	-45 ± 2	-51 ± 1	-47 ± 1
TEM Size [nm]	109 ± 13	90 ± 12	78 ± 12	74 ± 15
DLS Size [nm]	164 ± 3	155 ± 1	150 ± 2	147 ± 2
PDI	0.06	0.08	0.06	0.12

The MoO_2 NPs cores had a soft PVP coating that render them high colloidal stability in aqueous media. This polymeric soft coating also plays the role of a primer facilitating the SiO_2 coating on the surface of the clusters. In our earlier work,[13], we showed that Rh and Ru NPs with a PVP surface coating have the potential to be used as XFCT contrast agents, although their cytotoxicity profile showed some *in vitro* toxicity at higher concentrations. As a strategy to reduce toxicity, SiO_2 could represent a good material candidate for NP coating. As the Rh and Ru have the same PVP surface capping as MoO_2 NPs, the above-described novel method of SiO_2 coating with EA as the catalyst was assessed to be also applicable for the metallic NPs as platforms. In this respect, the same optimized EA and SiO_2 precursor (TEOS) concentrations used for MoO_2 NPs were applied to Rh and Ru NPs.

As there is a big difference in size between the MoO_2 clusters and Rh and Ru NPs, the concentration of core NPs was adapted to keep constant the ratio of the core-shell NPs diameter to the core NPs diameter. Hydrodynamic sizes of these NPs were estimated as $34(\pm 1)$ nm for Rh and $40(\pm 1)$ nm for Ru NP stock solutions (see table 1). The polydispersity index (PDI) of the DLS measurements (around 0.2) indicated slight agglomeration of the NPs, probably due to their low surface charge and/or to the PVP coating bridging several NPs in solution.[14] Surface area calculations, as detailed below, led to an optimal

concentration of 50 μg/mL for Rh and Ru NPs, to be used for further SiO_2 coating. The ζ-potential of the coated NPs indicated the successful coating of the core NPs, accompanied by a decrease in surface charge from close to the isoelectric point (IEP) for core NPs to strongly negative values for the coated NPs. The significant reduction in PDI values confirmed the uniform SiO_2 coating on both the metallic cores. The DLS measurements revealed that after coating the hydrodynamic (DLS) diameter ~ 100 nm for both core types. The TEM micrographs of SiO_2-Rh and SiO_2-Ru NPs (figure 1) showed a uniform SiO_2 coating with several metallic cores entrapped in one core-shell NP. The slight differences in core-shell architecture compared to single core-shell for MoO_2 can be explained by the very small core size for Rh and Ru as well as by a possible bridging effect by PVP in suspension of a small number of NPs.

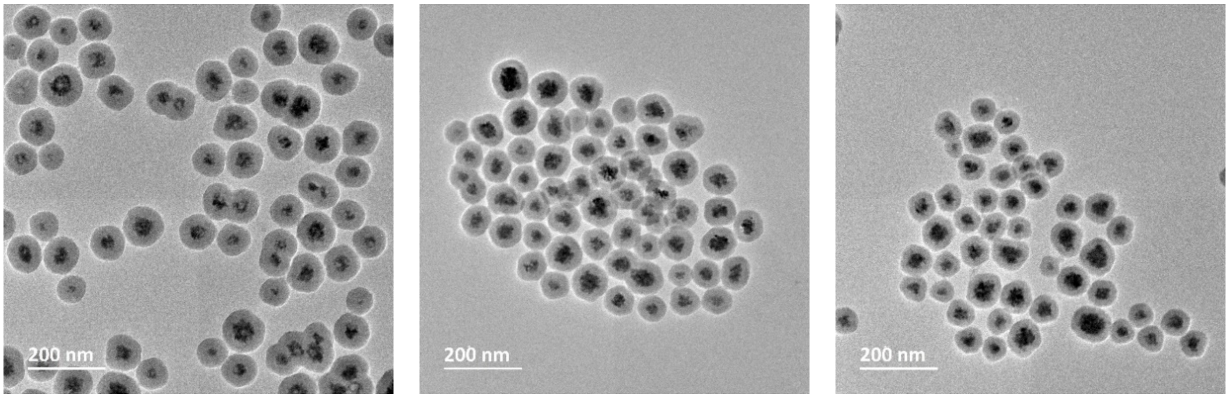

(a) **(b)** **(c)**

Figure S5. TEM micrographs of MoO_2-SiO_2 core-shell NPs, using three different concentrations of MoO_2 NPs as (**a**) 100 μg/mL, (**b**) 150 μg/mL, and (**c**) 200 μg/mL, while keeping constant the concentrations of the other precursors.

Surface area calculations

For the SiO_2-coating of Rh and Ru, the concentration of NPs was adapted in order to keep constant the ratio of total core-shell diameter to the core diameter calculated by the following formula:

$$\mathrm{R_{tot}} = \mathrm{R_{core}} + \mathrm{R_{shell}} = \mathrm{R_{core}} + k\mathrm{R_{core}} = (k+1)\mathrm{R_{core}},$$

where $\mathrm{R_{tot}}$ is the TEM radius of the core-shell NPs, $\mathrm{R_{core}}$ is the TEM core NPs radius and k is a quantity proportional to the inverse total surface area.

It is possible to infer that the quantity k will directly depend upon the concentration of NPs in the dispersion, assuming that all the other reaction parameters are kept constant and that the silicon source is infinite. The experimental work on the optimization of EA and silica shell thickness adjustment confirmed, that keeping constant all the other reaction parameters, by either changing the catalyst concentration even to a high concentration of 0.24 M or increasing the core concentrations 4 times from 50 μg/mL to 200 μg/mL, the silica did uniformly deposit on the core NPs. This is showing that for a concentration of 0.01 M TEOS and 2-hour reaction time at room temperature, the silicon precursor is in excess making our assumption that the silicon source is infinite correct.

From the formula above, to keep the ratio R_{tot}/R_{core} constant, the quantity k must be kept constant. Defining the total surface area S_{tot}, we can write:

$$k \propto \frac{1}{S_{tot}} = \frac{1}{S_{Np} \cdot N_{Np}} = \frac{\delta_{core}}{[NPs]} \cdot R_{core} \, ,$$

where S_{Np} is the NP surface area, N_{Np} is the number of NPs in the dispersion. Considering that:

$$S_{NP} = 4\pi \cdot R_{core}^2$$

and that

$$N_{NP} = [NPs_s]/m_{NP}$$

where [NPs] is the concentration of NPs and m_{NP} is the mass of a NP,
and that

$$m_{NP} = \frac{4}{3}\pi \cdot \delta_{core} R_{core}^3$$

where δ_{core} is the NPs density. The equation becomes:

$$k \propto \frac{\delta_{core}}{[NPs]} \cdot R_{core}$$

Thus, for MoO_2 with a δ_{core} of 6.47 g/cm³ for MoO_2 and a cluster R_{core} of $2.35 \cdot 10^{-6}$ cm² and a [NPs] of 0,150 g/L, we obtain:

$$\left.\frac{1}{S_{tot}}\right|_{MoOx} \approx 1 \frac{L}{m^2},$$

In order to obtain the same inverse total surface area k, the needed concentration for Rh and Ru is estimated to be:

$$[Rh] \approx [Ru] \approx 50 \text{ ppm},$$

considering δ_{core} of 12.41 and 12.45 g/cm^3 for Rh and Ru. Additionally, it is considered that Ru based core-shell NPs have 3 NPs per silica-coated cores so the R_{core} for Ru is 7.12 nm.

Cy5.5 Conjugation in the Silica Shell

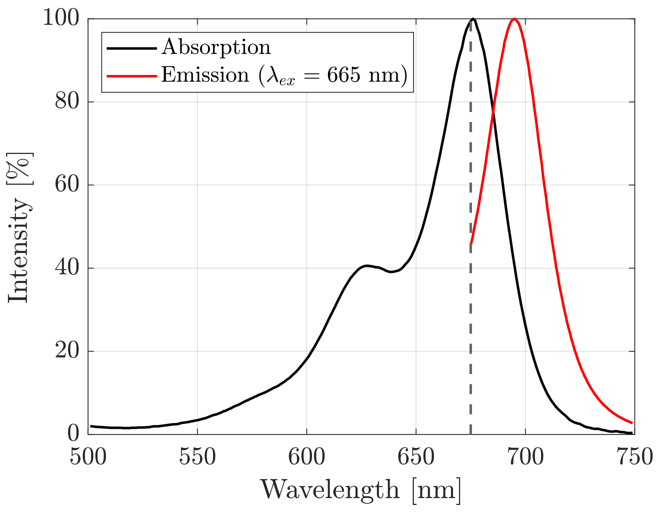

Figure S6. UV-Vis Absorption and PL spectra (excitation at 665 nm) of Cy5.5-APTES complex in DI water.

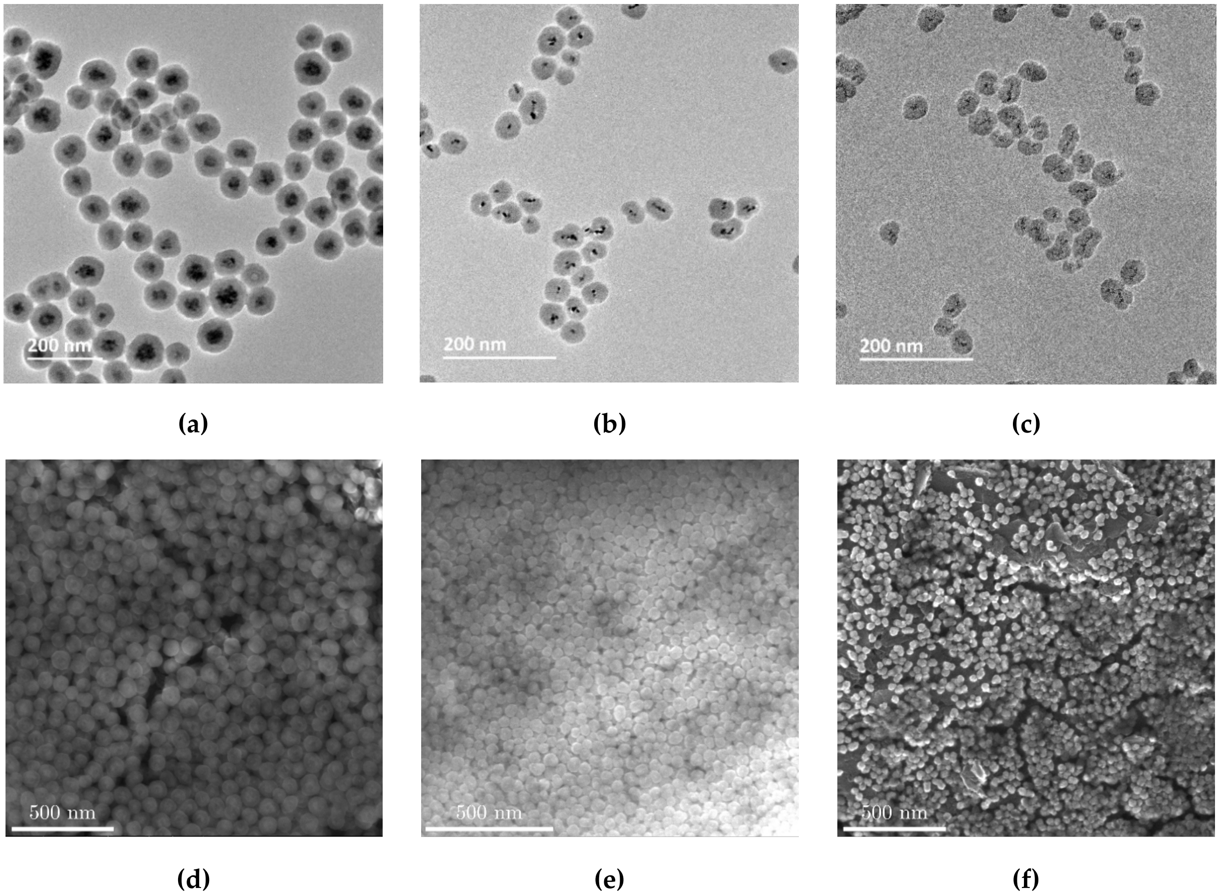

Figure S7. TEM and SEM micrographs for Cy5.5-conjugated core-shell NPs, using three different cores, (**a, d**) MoO_2, (**b, e**) Rh and (**c, f**) Ru NPs, respectively.

Table S3. ζ-Potential, TEM size, DLS size and PDI for Cy5.5-conjugated core-shell NPs; MoO_2-SiO_2-Cy5.5, Rh-SiO_2-Cy5.5 and Ru-SiO_2-Cy5.5 NPs.

	MoO_2-SiO_2-Cy5.5	Rh-SiO_2-Cy5.5	Ru-SiO_2-Cy5.5
ζ-Potential [mV]	-44 ± 2	-37 ± 2	-38 ± 2
TEM Size [nm]	79 ± 11	40 ± 9	38 ± 7
DLS Size [nm]	175 ± 5	109 ± 2	98 ± 3
PDI	0.18	0.20	0.21

In Vitro and *in Situ* Studies

Stability of silica coated core-shell nanoparticles in biological media has been previously studied, where it was shown that the stability of amorphous silica in the presence of potassium ions.[15] It is suggested that the formation of a potassium layer constitutes a shell to shield the silica from hydrolysis. The outstanding stability of silica nanoparticles in PBS was also supported in previous *in vitro* research.[16] In our work, we exposed the core-shell NPs to the cells in PBS, where the abundant potassium may form a protection layer around the silica shell. From the TEM observations, in Figure S8, we did not measure any significant change of the overall size of the core-shell NPs. This revealed a very slow kinetics of the dissolution process.

The evaluation of the potential toxicity of the newly developed NP is crucial if they are intended for clinical applications. It is also important to choose the relevant toxicity models with potential to predict toxicity *in vivo*. As a first step we have evaluated NP toxicity *in vitro* on a murine macrophage cell line since macrophages are intrinsic components of all organs contributing to the tissue's homeostasis. Additionally, the macrophages are scavengers that are rapidly recruited to organs following external insult (ex, NP accumulation), to engulf and clear out foreign materials. Though the translation of the results from *in vitro* to *in vivo* settings is difficult, the murine origin of the cell line facilitates comparison to *in vivo* studies in rodents.

The RAW 264.7 are adherent cells and Real-time Cell Analysis (RTCA) can accurately monitor their behavioral changes in real-time without any intervention necessary during the experiments (medium changes, addition of labels), which may interfere with cell function. In this assay, the increments in cell confluence will result in a proportional impedance of the electric current flow, through electrodes integrated in the bottom of the cell culturing plate. The impedance readout is an arbitrary value expressed as cell index (CI). Any factor that affects cell adherence (*e.g.* blocked proliferation, or morphology changes) would reflect in fluctuations in impedance value.[17] This technology is widely used for drug screening to determine the cytotoxic potency effects of pharmacological agents.[18,19]

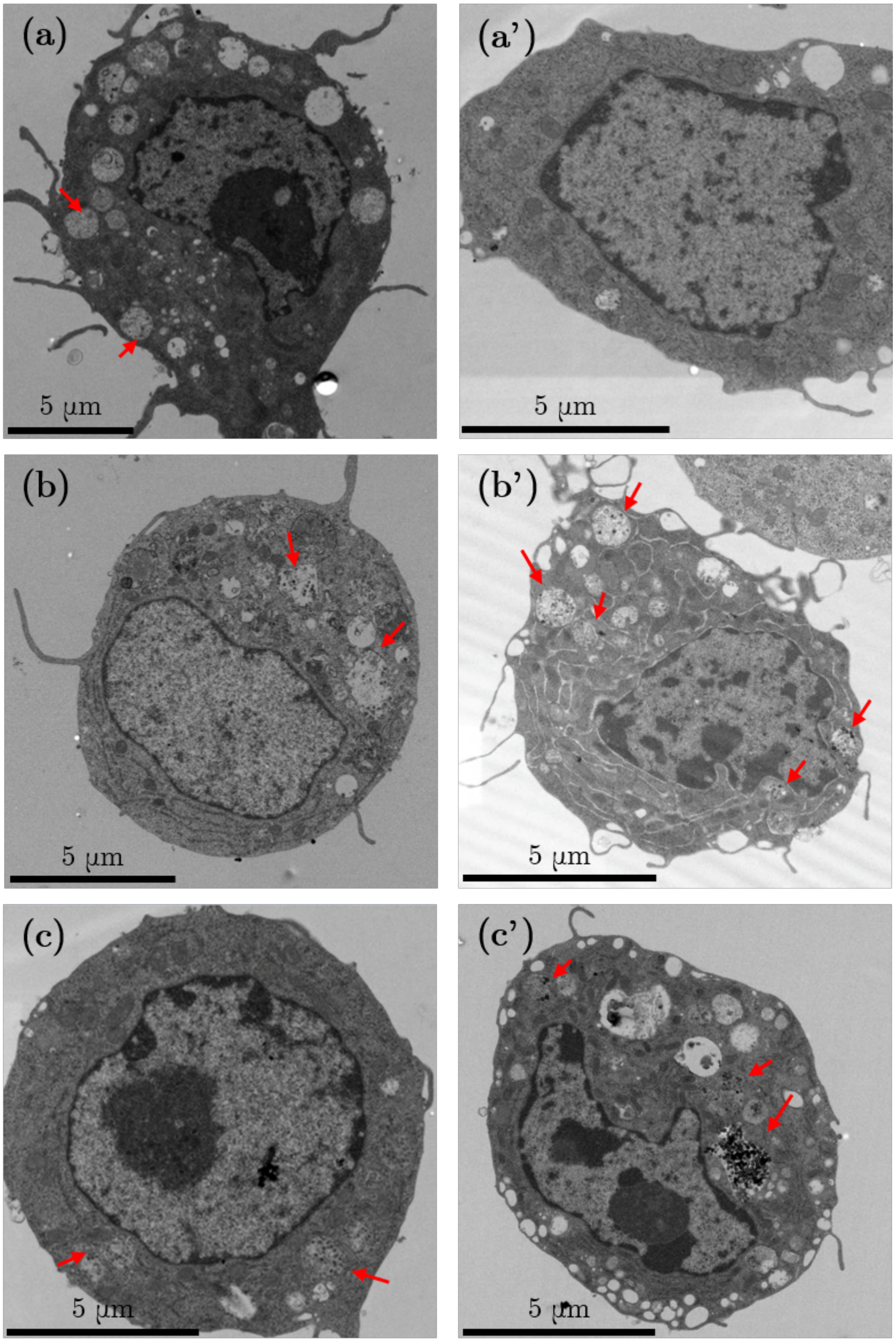

Figure S8 TEM micrographs of RAW 264.7 incubated with MoO_2 (**a**, **a′**), MoO_2-SiO_2 (**b**, **b′**) and MoO_2-SiO_2-Cy5.5 (**c**, **c′**) NPs for 2 h (**a**, **b** and **c**) and for 24 h (**a′**, **b′**, **c′**). The red arrows indicate the localization of the NPs in cells.

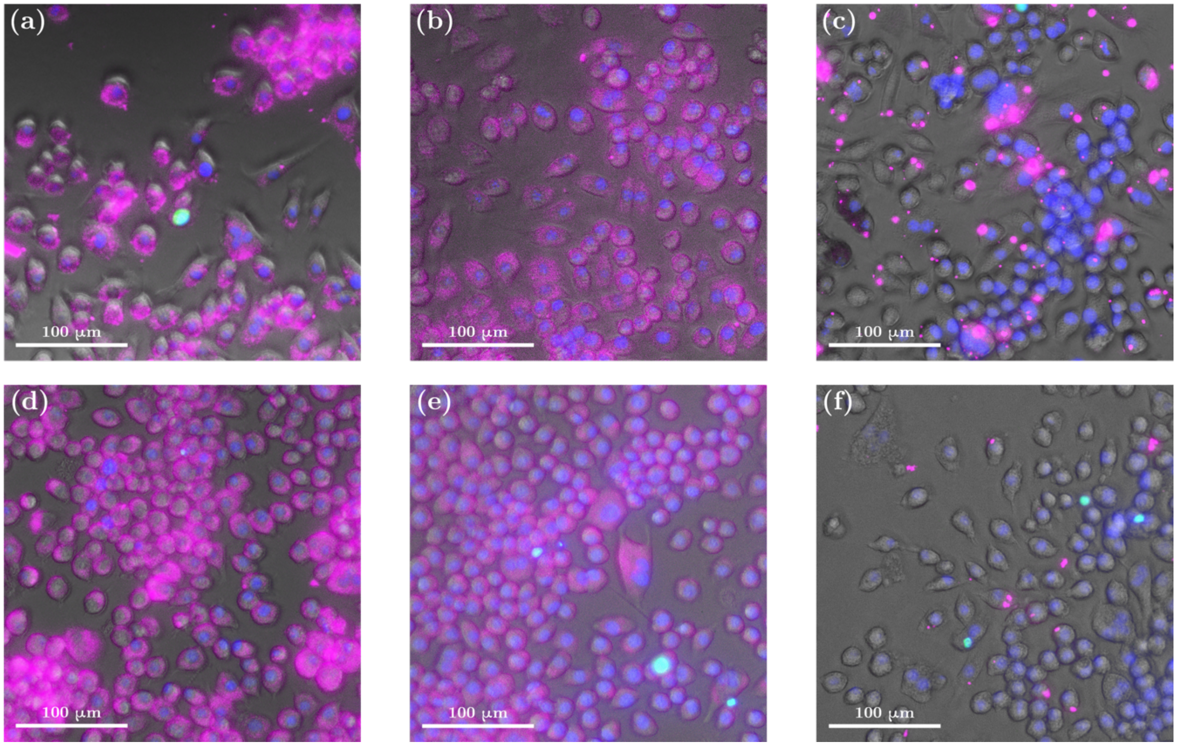

Figure S9. Live images of RAW Macrophages incubated with the Cy5.5-conjugated samples (250 μg/mL, in pink) with three different cores, (a-d) MoO_2, (b-e) Rh, and (c-f) Ru, and different incubation times, 24 h (a, b, c) and 72 h (d, e, f). DAPI (in blue) and NucGreen (in green) are markers for cell nuclei and dead cells respectively. Trans-luminescence signal is included to highlight cell morphology.

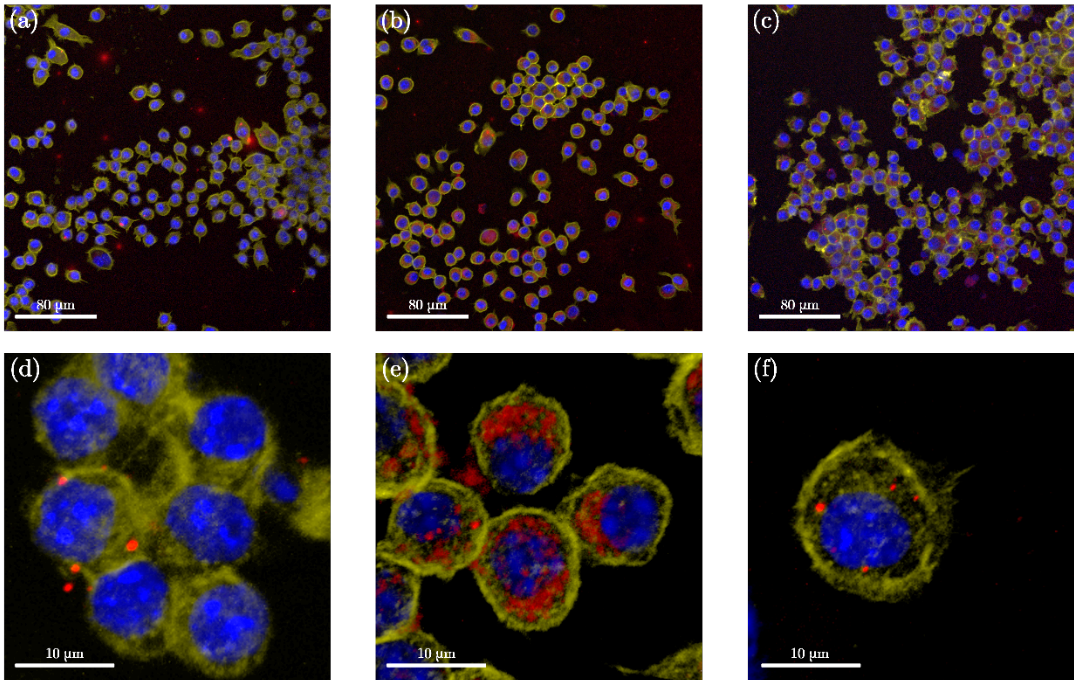

Figure S10. Confocal images of fixed and stained RAW Macrophages incubated for 72 h with the Cy5.5-doped samples (250 μg/mL, in red) with three different cores, (a-d) MoO_2, (b-e) Rh and (c-f) Ru, at different magnifications (20x and 63x). DAPI (in blue) and Alexa 555 - Phalloidin (in yellow) are markers for cell nuclei and actin filaments, respectively.

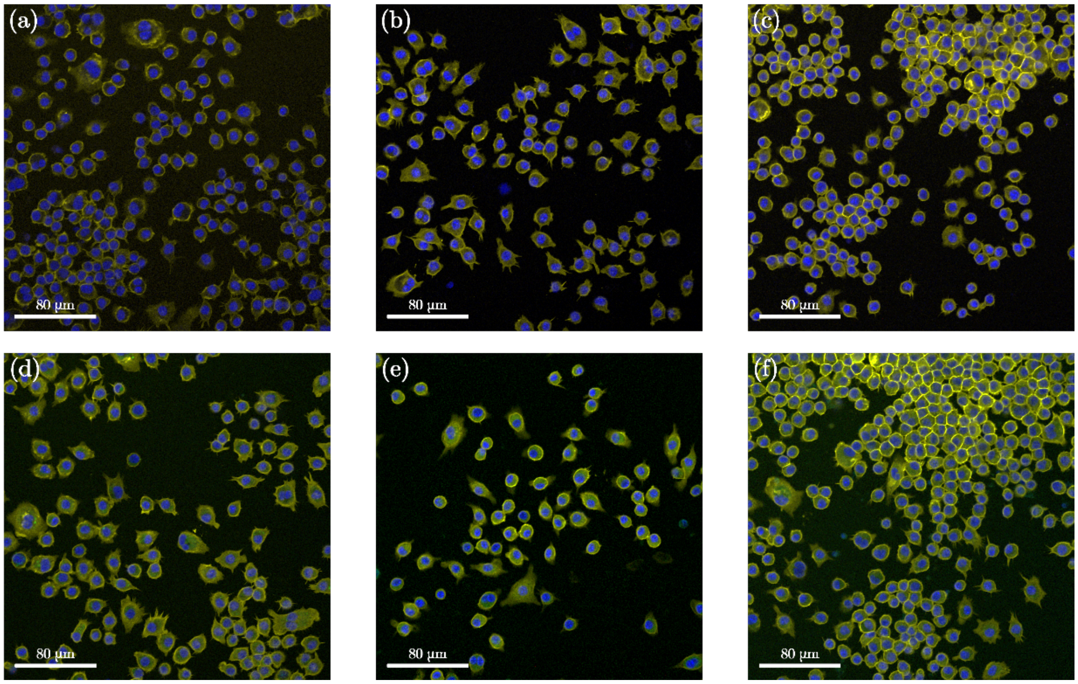

Figure S11. Confocal images of fixed and stained RAW264.7 Macrophages incubated with core- SiO_2 shell NP samples (250 μg/mL) without Cy5.5 with three different cores, (a-d) MoO_2, (b-e) Rh and (c-f) Ru, at different incubation times, 24 h (a, b, c) and 72 h (d, e, f). DAPI (in blue) and Alexa 555 - Phalloidin (in yellow) are markers for cell nuclei and actin filaments, respectively.

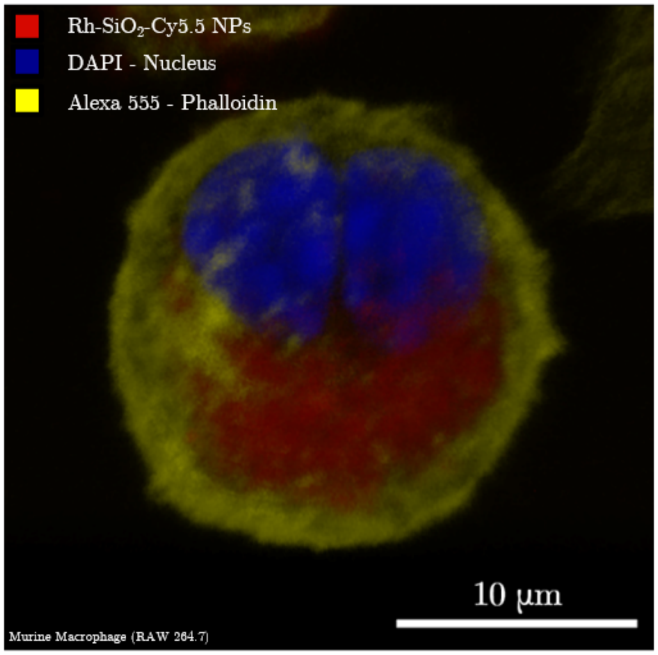

Figure S12. A fixed RAW Macrophage incubated with Rh-SiO_2-Cy5.5 NPs (250 μg/mL, in red), imaged with confocal microscopy (deconvoluted image, 63x). DAPI (in blue) and Alexa 555 - Phalloidin (in yellow) are markers for cell nuclei and actin filaments, respectively.

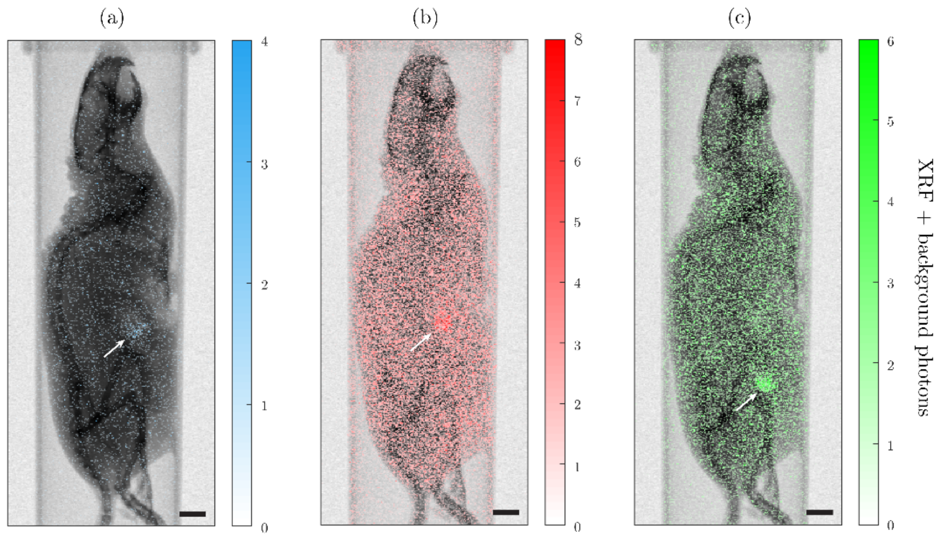

Figure S13. Example of raw XRF projection images (color) overlaid on top of X-ray absorption projections (grayscale). The raw XRF projection images are extracted from the XFCT detectors by selecting a spectral range corresponding to the different elements of interest (cf. Figure 6d, main text), here (a) Mo (blue, [17.1 - 17.7] keV), (b) Rh (red, [19.9 - 20.4] keV), (c) and Ru (green, [18.9 - 19.6] keV). The ranges were optimized for maximum signal-to-background (SBR) ratio. The 3 mm spherical sample holders with MoO_2-SiO_2-Cy5.5, Rh-SiO_2-Cy5.5 and Ru-SiO_2-Cy5.5 NPs respectively can be identified as regions with higher signal (arrows). Color bars denote the number of recorded X-ray photons in the spectral ranges chosen, which is the sum of XRF and background photons from Compton scattering. The maximum of each color bar corresponds to the maximum number of recorded photons in each projection image, showcasing that while XRF signal levels are similar for the different core-element NPs, the background contribution is highest for Rh, followed by Ru and last Mo. The different background contribution is ascribed to the presence of Compton scattering, determined by the energy of the X-ray source. A source with higher energy would lead to a shift of the Compton scatter, thus decreasing the background contribution, but with lower signal due to a greater energy mismatch with the K-absorption edge of the NPs. For tomographic reconstruction, the background contribution seen here is estimated and subtracted from each projection image prior to further processing. The background was estimated by removing signal from all pixels until the observed signal outside the mouse (e.g., scattering in the animal holder) was removed. This resulted in a per-pixel background estimate of up to 1 photon for Mo, 2 photons for Ru and 3 photons for Rh. Scalebars: 5 mm.

References

(1) Zeng, X.; Zhou, B.; Gao, Y.; Wang, C.; Li, S.; Yeung, C. Y.; Wen, W. J. Structural Dependence of Silver Nanowires on Polyvinyl Pyrrolidone (PVP) Chain Length. *Nanotechnology* **2014**, *25* (49), 495601. https://doi.org/10.1088/0957-4484/25/49/495601.

(2) Brinker, C. J.; Scherer, G. W. *Sol-Gel Science: The Physics and Chemistry of Sol-Gel Processing*; Academic Press, Inc., San Diego, **1990**. https://doi.org/10.1016/C2009-0-22386-5.

(3) Bogush, G. H.; Tracy, M. A.; Zukoski IV, C. F. Preparation of Monodisperse Silica Particles: Control of Size and Mass Fraction. *J. Non-Cryst. Solids* **1988**, *104* (1), 95–106. https://doi.org/10.1016/0022-3093(88)90187-1.

(4) Lismont, M.; Páez, C. A.; Dreesen, L. A One-Step Short-Time Synthesis of Ag@SiO_2 Core-Shell Nanoparticles. *J. Colloid Interface Sci.* **2015**, *447*, 40–49. https://doi.org/10.1016/j.jcis.2015.01.065.

(5) Hull, M. N. On the Anodic Dissolution of Molybdenum in Acidic and Alkaline Electrolytes. *J. Electroanal. Chem.* **1972**, *38* (1), 143–157. https://doi.org/10.1016/S0022-0728(72)80100-1.

(6) Badawy, W. A.; Al-Kharafi, F. M. Corrosion and Passivation Behaviors of Molybdenum in Aqueous Solutions of Different pH. *Electrochim. Acta* **1998**, *44* (4), 693–702. https://doi.org/10.1016/S0013-4686(98)00180-7.

(7) Wang, L.; Zhang, G. H.; Chou, K. C. Study on Oxidation Mechanism and Kinetics of MoO_2 to MoO_3 in Air Atmosphere. *Int. J. Refract. Met. Hard Mater.* **2016**, *57*, 115–124. https://doi.org/10.1016/j.ijrmhm.2016.03.001.

(8) Krishnan, C. V.; Garnett, M.; Hsiao, B.; Chu, B. Electrochemical Measurements of Isopolyoxomolybdates: 1. pH Dependent Behavior of Sodium Molybdate. *Int. J. Electrochem. Sci.* **2007,** 2, 29 - 51.

(9) Roth, K. M.; Zhou, Y.; Yang, W.; Morse, D. E. Bifunctional Small Molecules Are Biomimetic Catalysts for Silica Synthesis at Neutral pH. *J. Am. Chem. Soc.* **2005**, *127* (1), 325–330. https://doi.org/10.1021/ja045308v.

(10) Meier, M.; Ungerer, J.; Klinge, M.; Nirschl, H. Synthesis of Nanometric Silica Particles *via* a Modified Stöber Synthesis Route. *Colloids Surf., A* **2018**, *538*, 559–564. https://doi.org/10.1016/j.colsurfa.2017.11.047.

(11) Barisik, M.; Atalay, S.; Beskok, A.; Qian, S. Size Dependent Surface Charge Properties of Silica Nanoparticles. *J. Phys. Chem. C* **2014**, *118* (4), 1836–1842. https://doi.org/10.1021/jp410536n.

(12) Jelinek, L.; Dong, P.; Rojas-Pazos, C.; Kováts, E. S.; TaÏbi, H. Study of the Stöeber Reaction. 1. Properties of Colloidal Silica Spheres Prepared *via* Alkoxide Hydrolysis. *Langmuir* **1992**, *8* (9), 2152–2164. https://doi.org/10.1021/la00045a015.

(13) Li, Y.; Shaker, K.; Svenda, M.; Vogt, C.; Hertz, H. M.; Toprak, M. S. Synthesis and Cytotoxicity Studies on Ru and Rh Nanoparticles as Potential X-Ray Fluorescence Computed Tomography (XFCT) Contrast Agents. *Nanomaterials* **2020**, *10* (2), 310. https://doi.org/10.3390/nano10020310.

(14) McFarlane, N. L.; Wagner, N. J.; Kaler, E. W.; Lynch, M. L. Poly(Ethylene Oxide) (PEO) and Poly(Vinyl Pyrolidone) (PVP) Induce Different Changes in the Colloid Stability of Nanoparticles. *Langmuir* **2010**, *26* (17), 13823–13830. https://doi.org/10.1021/la101907s.

(15) Wynn, T. A.; Chawla, A.; Pollard, J. W. Macrophage Biology in Development, Homeostasis and Disease. *Nature* **2013**, 445–455. https://doi.org/10.1038/nature12034.

(16) Dos Santos, T.; Varela, J.; Lynch, I.; Salvati, A.; Dawson, K. A. Quantitative Assessment of the Comparative Nanoparticle-Uptake Efficiency of a Range of Cell Lines. *Small* **2011**, *7* (23), 3341–3349. https://doi.org/10.1002/smll.201101076.

(17) Tripathy, N.; Hong, T. K.; Ha, K. T.; Jeong, H. S.; Hahn, Y. B. Effect of ZnO Nanoparticles Aggregation on the Toxicity in RAW 264.7 Murine Macrophage. *J. Hazard. Mater.* **2014**, *270*, 110–117. https://doi.org/10.1016/j.jhazmat.2014.01.043.

(18) Carlander, U.; Midander, K.; Hedberg, Y. S.; Johanson, G.; Bottai, M.; Karlsson, H. L. Macrophage-Assisted Dissolution of Gold Nanoparticles. *ACS Appl. Bio Mater.* **2019**, *2* (3), 1006–1016. https://doi.org/10.1021/acsabm.8b00537.

(19) Giaever, I.; Keese, C. R. Use of Electric Fields to Monitor the Dynamical Aspect of Cell Behavior in Tissue Culture. *IEEE Trans. Biomed. Eng.* **1986**, *BME-33* (2), 242–247. https://doi.org/10.1109/TBME.1986.325896.

(20) Atienzar, F. A.; Tilmant, K.; Gerets, H. H.; Toussaint, G.; Speeckaert, S.; Hanon, E.; Depelchin, O.; Dhalluin, S. The Use of Real-Time Cell Analyzer Technology in Drug Discovery: Defining Optimal Cell Culture Conditions and Assay Reproducibility with Different Adherent Cellular Models. *J. Biomol. Screen.* **2011**, *16* (6), 575–587. https://doi.org/10.1177/1087057111402825.

(21) Ke, N.; Wang, X.; Xu, X.; Abassi, Y. A. The XCELLigence System for Real-Time and Label-Free Monitoring of Cell Viability. *Methods Mol. Biol.* **2011**, *740*, 33–43. https://doi.org/10.1007/978-1-61779-108-6_6.
